# Intuitions of mathematical curves in young children’s drawings

**DOI:** 10.1016/j.cognition.2025.106359

**Published:** 2025-11-01

**Authors:** Lorenzo Ciccione, Marie Lubineau, Théo Morfoisse, Stanislas Dehaene

**Affiliations:** a Cognitive Neuroimaging Unit, CEA, INSERM, Université Paris-Saclay, NeuroSpin Center, 91191 Gif/Yvette, France; b Department of Psychology, DysCo Lab, Université Paris 8, 93526 Saint-Denis, France; c Collège de France, Université Paris Sciences Lettres (PSL), 11 Place Marcelin Berthelot, 75005 Paris, France

**Keywords:** Geometric patterns, Proto-mathematics, Child development, Pattern recognition, Compositional reasoning, Drawing

## Abstract

How sophisticated is young children’s comprehension of geometric lines, curves and patterns, and how can we probe it? We investigated early proto-mathematical intuitions by asking kindergarteners (*N* = 39, 25 girls, 66 months) and first-graders (*N* = 42, 20 girls, 76 months) to draw the prolongation of mathematical patterns. Children’s drawings revealed an early yet partial understanding of key mathematical properties such as linearity, curvature, periodicity, and compositionality. These abilities were confirmed in a second task, where participants were asked to select the correct prolongation among six options, ruling out motor entrainment as an explanation for the drawing task. These findings highlight children’s emerging intuitions of proto-mathematical concepts and underscore the potential of drawing as a powerful and concrete tool for assessing early mathematical reasoning.

## 1 Introduction

Since the dawn of time, humanity has engaged in the representation of abstract geometric symbols, such as when *Homo erectus* carved parallel lines and zigzags on a shell in Java around 540,000 years ago (Joordens et al., 2015). These early graphic productions can be defined in today’s terminology as geometric symbols or spatial patterns – i.e., dots, lines, curves and objects arranged according to specific rules. Despite their ancient origins, patterns continue to play an important role in today education, as most preschools employ a variety of pattern-based activities, such as arranging blocks of alternating colors, repeating nursery rhymes, and drawing symmetrical objects (Cohen & Emmons, 2017; Gluschankof, 2008; Villarroel, Merino, & Antón, 2018). While some educators view such activities as merely motor or creative tasks, they actually engage deeper mathematical concepts. Indeed, in order to understand and manipulate patterns, one must organize, memorize, and compress information by using, at least implicitly, geometrical features such as symmetry, parallelism, angles and length (Dehaene, Al Roumi, Lakretz, Planton, & Sablé-Meyer, 2022; Mills, Tenenbaum, & Cheyette, 2023; Sablé-Meyer, Ellis, Tenenbaum, & Dehaene, 2022). For these reasons, mathematics has been defined as “the science of patterns” (Steen, 1988). Because the comprehension of patterns seems to emerge before or even in the absence of any training or formal education, they are best viewed as “proto-mathematical” in nature. Based on this view of patterns as proto-mathematical objects, some cognitive scientists have postulated that stimulating abstract thinking through pattern recognition and reproduction could boost the acquisition of more formal mathematical and algebraic reasoning (Orton, 2004; Zippert, Douglas, & Rittle-Johnson, 2020). However, this hypothesis has received only mixed support so far, with some empirical studies showing a correlation between pattern understanding and academic achievement in mathematics (Kidd et al., 2014; Lee, Ng, Bull, Pe, & Ho, 2011; Pasnak, 2017; Vanluydt, Wijns, Torbeyns, & Van Dooren, 2021; Wijns, Verschaffel, De Smedt, & Torbeyns, 2021) and others finding no such correlation (Wijns, Verschaffel, De Smedt, De Keyser, & Torbeyns, 2021).

Even if the exact link between mathematical skills and pattern understanding remains unclear, humans undeniably hold intuitions about patterns from a very young age, before any formal schooling in mathematics. Around the age of 3, for example, children are able to copy 3D constructions of 3 colored blocks (Verdine et al., 2014). By 4 years old, they are capable of extending and generalizing several types of patterns, such as transposing series of two blue and two red squares into series of two green and two yellow triangles, although they still have difficulties identifying the minimal unit that is being repeated (Rittle-Johnson, Fyfe, McLean, & McEldoon, 2013). Furthermore, children’s performance increases significantly if they are encouraged to focus on the abstract nature of the configurations presented to them (Fyfe, McNeil, & Rittle-Johnson, 2015). For example, if three objects such as a blue, a red and a blue cube are associated with letter labels A, B, A, this manipulation facilitates children’s transposition of the pattern to different items (such as orange, yellow and orange pyramids).

However, studying pattern understanding with discrete objects, as described so far, has a limitation: those materials do not allow to investigate the abstraction of more complex mathematical rules. Let us take the case of colored bricks or musical notes following a growing pattern (e.g., AB AABB AAABBB). Extrapolating the extension of such a series can be limited by children’s working memory capacities or by the complexity of the task, especially if the pattern follows complex rules. Failing such a task would not necessarily reflect a lack of understanding, but rather an overload of working memory or a difficulty in understanding the task. Various studies seem to confirm that difficulties with visual patterns of this kind persist even into adolescence (MacGregor & Stacey, 1995; Warren, 2005), thus discouraging the indiscriminate use of patterns based on discrete items for educational purposes.

Given these considerations, how can we study children’s skills at recognizing and understanding complex patterns? One promising approach involves using mathematical displays such as curves and graphs, which help overcome many of the limitations discussed above, as they can represent complicated patterns as a single object (Mills et al., 2023, 2024). For example, the graph of a linear function visually expresses an increasing pattern, while the graph of a sinusoidal function captures an alternating pattern. Such representations offer access to a wide range of patterns that would be hard to convey with discrete objects (although see de Hevia & Spelke, 2010 and de Hevia, Vanderslice, & Spelke, 2012 for clever visual representations of a linear increase accessible even to infants).

A key advantage of representing patterns as mathematical functions and geometric motifs is to allow for the composition of primitive functions into more complex composite curves. A zigzag with an increasing peak-to-peak amplitude, for instance, can be seen as a pattern that combines an oscillation or alternation (zig-zag) with a linear trend on one of its parameters (amplitude). Such a layered structural interpretation aligns with cognitive theories proposing that human symbolic drawings reflect a compositional, recursive language-of-thought that can recombine elementary operations (e.g., drawing a line) into more complex programs (e.g., drawing a square, or even a square of circles; Amalric et al., 2017; Dehaene, 2026; Dehaene et al., 2022; Mills et al., 2023; Piantadosi, Tenenbaum, & Goodman, 2016; Schulz, Tenenbaum, Duvenaud, Speekenbrink, & Gershman, 2017). Compositionality can be defined as the capacity to form complex mental representations through the combination of several preexisting ones (Frankland & Greene, 2020; Lake, Ullman, Tenenbaum, & Gershman, 2016; Schulz et al., 2017). According to the language of thought hypothesis (Amalric et al., 2017; Dehaene et al., 2022; Frankland & Greene, 2020; Mills et al., 2023; Piantadosi et al., 2016; Schulz et al., 2017), human adults are endowed with the peculiar skill of representing complex concepts by combining elementary ones through compositional operations of recursion, concatenation and repetition. Whether and when compositional geometric programs are available to young children is an open question (Coates, Siegel, Tenenbaum, & Schulz, 2023; Mills et al., 2024; Morfoisse et al., 2025; Sablé-Meyer et al., 2021), which we begin to explore here by asking whether they understand composite patterns such as a zigzag or sinusoid of increasing oscillation amplitude.

To investigate how children understand such visual patterns and uncover their proto-mathematical intuitions, we developed two complementary tasks. The first and main experiment, stems from a rich tradition in developmental psychology (Goodenough, 1928; Good-enough & Harris, 1950; Luquet, 1927), where children’s drawings are used to probe cognitive development. While previous research focused on free drawing (Dillon, 2021; Lange-Küttner, 2014; Long, Fan, Huey, Chai, & Frank, 2024; Morfoisse, Gureckis, & Dillon, 2020), we introduced here a more structured task that can be described as “constrained drawing”, centered on a very specific class of objects: mathematical functions and patterns. On each trial, we show a continuous curve on the left side of a sheet of paper and ask children to first trace it with a felt-tip pen, then continue it by drawing a prolongation on the right side of the page. By varying the patterns that are proposed for extrapolation, we can study the comprehension of a broad range of proto-mathematical concepts.

This drawing task also allows us to keep the space of possible responses fully open, without imposing any strong expectations or prior assumptions. However, drawing performance may also be influenced by motor abilities. Success in extrapolating a curve might reflect motor entrainment rather than conceptual understanding, and conversely, some errors could stem from motor difficulties rather than cognitive limitations. To disentangle these possibilities, we designed a second experiment using a multiple-choice task. A new group of children was shown initial curves and asked to choose the correct continuation from six options: a first curve labelled as correct, following the mathematical expression used to draw the initial part of the curve, and five others violating specific properties such as continuity, derivability or curvature. The drawing task (experiment 1) and the multiple-choice task (experiment 2) therefore were complementary: while multiple-choice imposes a selection of distractors, unlike the drawing task, it helps to isolate pattern comprehension from motor execution and provides a more controlled measure of children’s understanding.

At present, there is only limited but growing evidence that kinder-garteners and first graders may already understand geometric concepts and their combinations (Amalric et al., 2017; Gao & Hu, 2024; Huey, Jordan, Hart, & Dillon, 2023; Izard, Pica, & Spelke, 2022; Izard & Spelke, 2009; Morfoisse et al., 2025; Sablé-Meyer et al., 2021). As concerns graphic displays of mathematical functions, children are able to quickly extract the increasing versus decreasing trend of linear patterns, similarly to adults (Ciccione et al., 2023), thus suggesting a certain degree of intuition about graphical representations. In addition, drawing is an activity particularly enjoyed by children, even by the youngest ones (Forkosh & Drake, 2017), and already present at school. It is common to observe children prolonging borders or curves according to a predefined pattern, a game that is often seen by teachers as propaedeutic to the development of creativity and motor skills. However, to our knowledge, no formal investigation of such drawing skills has been proposed. We therefore decided to capitalize on all these aspects to address several research questions:

1) **Do children’s drawing abilities reveal a significant understanding of complex patterns?** Thanks to the versatility that the drawing method provides, we can study the ability to extrapolate mathematical functions such as quadratics and sinusoids (Ciccione & Dehaene, 2021; Ciccione, Sablé-Meyer, & Dehaene, 2022; Schulz et al., 2017; Schulz, Tenenbaum, Reshef, Speekenbrink, & Gershman, 2015), which would be difficult to represent using classic tools. If kindergartners and first graders succeed at this task, this would strongly suggest that the knowledge of functional relationships in visual patterns is available early on in development. This would also provide a proof of concept for the investigation of the comprehension of complex patterns via the drawing method in young learners.
2) **How does pattern understanding evolve during child development?** To explore this question, we decided to test both kindergarteners and first graders, examining their abilities just before and just after the beginning of formal learning of mathematics and reading. Testing these two populations should shed some light on whether and how the catalogue of geometric functions gets enlarged during development.
3) **Are children able to understand and extrapolate composite patterns?** Here, we examine whether the drawing task provides a window to the study of compositionality, by analyzing the prolongation of curves that combine several types of changes – for instance a zigzag with increasing peak-to-peak amplitude. We tested to what extent children understand that this curve results from the composition of the primitives involved: the elementary line segments, their periodic recurrence, and the overall increase in peak-to-peak amplitude. It is possible that children (especially kindergartners) would only extract one primitive (e.g., the periodic nature of the pattern), thus failing to correctly compose all functional primitives.
4) **Are drawing results driven by motor components, and can they be replicated in a more controlled perceptual task?** To address this, we conducted a second experiment using a multiple-choice task, where children were shown initial curves and asked to choose the correct continuation from six options—one drawn from the mathematical function supporting the initial part of the curve and five violating core properties (e.g., continuity). This approach offers a motor-free complement to the drawing task, helping us assess whether the observed patterns stem from abstract understanding rather than motor execution.

## 2 Experiment 1

### 2.1 Methods

#### 2.1.1 Participants

We tested 43 kindergarteners (25 girls, average age: 66 months) and 39 first graders (20 girls, average age: 76 months) from 4 different Parisian schools. Given the new methodology employed and the exploratory nature of the study, the sample size was chosen by convenience and not as a result of a power analysis. Parents were informed of the purpose of our study and of the possibility for them to object to their child’s participation via a form given to the child by the teacher. Participants were tested by our research team during school time, in a quiet room close to the classroom.

#### 2.1.2 Experimental procedure

**Fig. 1:**
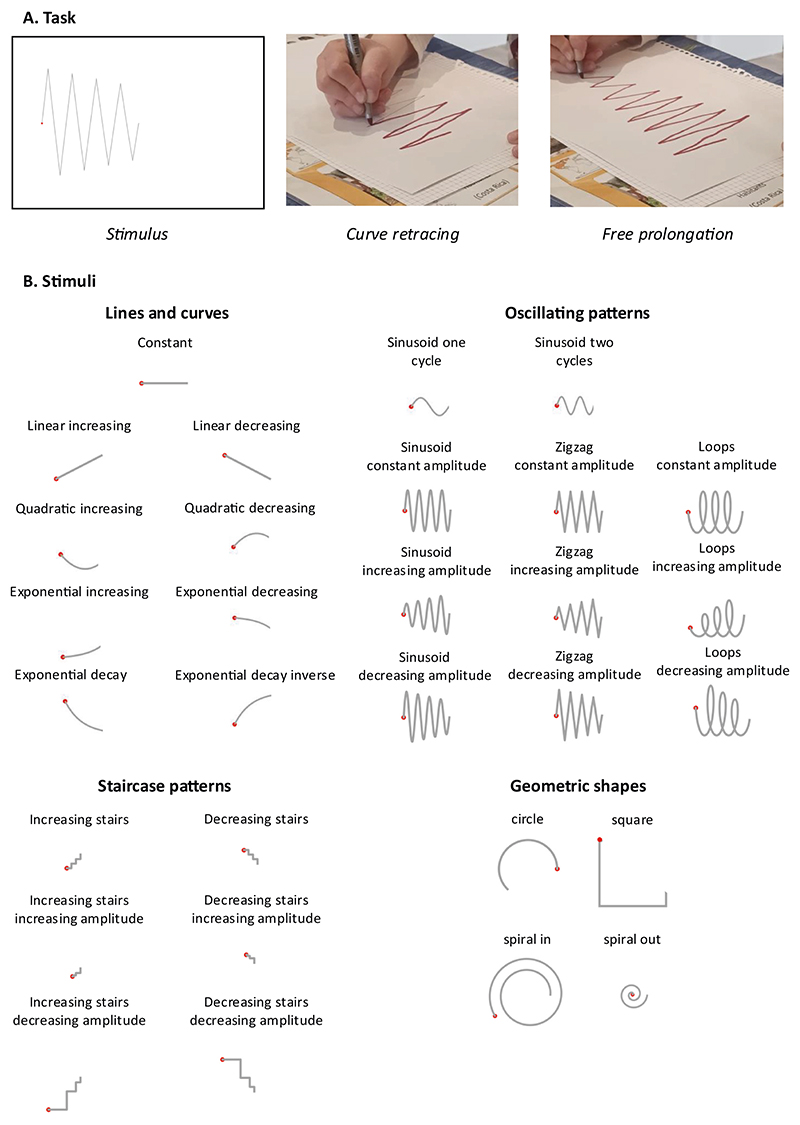
Experimental procedure (experiment 1). A, Task. On each trial, a stimulus curve was shown to the child, who was asked to first retrace it and then prolong it. B, Stimuli. 30 stimuli were presented. The constant line was always shown first as training, followed by all other patterns in random order (except the four geometric shapes, shown at the end).

Task and stimuli are presented in Fig. 1. Children were successively presented with 30 white A4 papers, on which a thin gray line occupied a portion of the left half. All patterns started with a red dot and ended at the same point, corresponding to the center of the sheet. Children were asked to redraw (with a red felt-tip pen) the pattern from the red dot and to prolong each pattern on the right half of the sheet.

The test always began with the constant linear function (a horizontal line segment), which served as a training exercise. The presentation of this first function was accompanied by the following instructions: “Today, we’re going to draw paths. A path always begins with a red dot and a gray line. Look closely at this path, can you see how it goes?” First, the experimenter showed the training path to the child, without a pen, so that the child could take time to observe its specific features. The experimenter then gave the child the pen and continued: “I’m going to ask you to put your felt-tip pen on this red dot, go over the gray line and then continue the path”. The child was then led by the experimenter to apply this instruction. If they misunderstood the instruction for this training exercise, the experimenter corrected the child, who was then given a second chance, with the same constant linear function. Our instructions deliberately used the word “path” (*chemin* in French) in order to induce a spatial understanding of the task, given prior results suggesting that this vocabulary induces spatial navigation strategies (Dillon, 2021; Lin & Dillon, 2023; Morfoisse et al., 2020). Although we did not formally test this, using terms like *“object”* or *“shape”* might have encouraged participants to close the figure rather than extend it from left to right as we intended (Lin & Dillon, 2023).

A total of 30 patterns (Fig. 1) were presented. Most of them (26) corresponded to graphs of mathematical functions whose variations allowed us to probe children’s implicit understanding of various mathematical properties such as position, slope, curvature, or periodicity. In addition to the constant line which was always used as training, the other 25 mathematical functions, presented in random order, comprised: (A) non-oscillating functions, including linear, quadratic, exponential increase, and exponential decay; all were presented with or without vertical flipping (mathematically, preceded by a plus or minus sign); (B) oscillating functions, including two sinusoids of constant amplitude but with different frequencies; sinusoids, zigzags and loops of constant frequency, presented in three different forms: constant peak-to-peak amplitude, decreasing peak-to-peak amplitude and increasing peak-to-peak amplitude; the amplitude of the last period was the same for all oscillating functions; (C) three stairs-like ascending functions: one of constant amplitude and frequency, one of increasing amplitude and frequency and one of decreasing amplitude and frequency (as well as their descending counterparts).

In addition to these functions, for exploratory purposes, we also included 4 partial geometric shapes (a portion of a circle, a portion of a square and two partial spirals), which were presented in a random order at the end of the experiment.

#### 2.1.3 Qualitative analysis

**Fig. 2:**
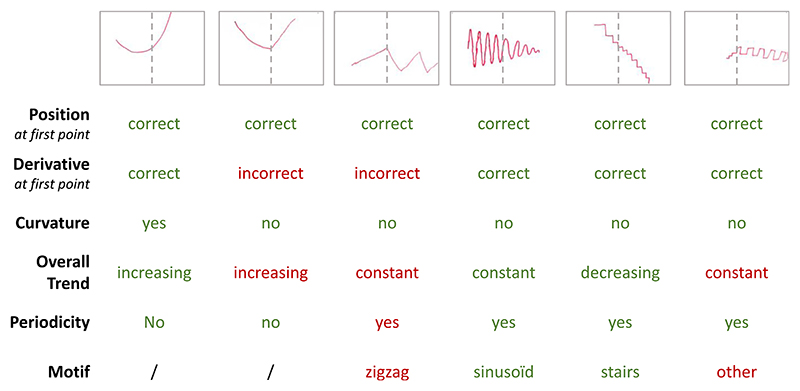
Qualitative analysis of children’s responses. Examples of children productions and answers given by the raters to the different questions. Green color indicates correct functional properties, whereas red color indicates wrong ones. (For interpretation of the references to color in this figure legend, the reader is referred to the web version of this article.)

To assess children’s productions as objectively as possible, 2 human raters each evaluated half of the drawings, and answered the following questions: (1) Position: At the point where extrapolation begins (past the middle of the page), is the pen in the right place (position)? (2) Derivative: Is the derivative of the trajectory a correct prolongation of the pattern on the left? (3) Curvature: Is the trajectory curved upwards, downwards, or is it straight? (4) Trend: What is the general trend of the extrapolated curve: increasing, constant, or decreasing? (5) Oscillation: Is the prolongation monotonic or oscillating? (6) Motif: If oscillating, what is the pattern’s motif: sinusoidal, zigzag, stairs, loop, or other? Fig. 2 provides examples of several drawings and, for each of them, the raters’ answers to those questions. All questions were answered using a computer form.

In order to make the raters blind to the expected answers, each drawing was first folded in half (except for the four geometrical shapes), so that only the right-hand side drawn by the child was visible. Raters first answered each question related to global parameters (curvature, overall trend, oscillating, motif). To answer the two questions related to local parameters (position and derivative), the rater finally unfolded the sheet, to see the actual pattern and make a judgment. For geometric shapes, raters only made a judgment on the correctness of the child production.

To measure inter-raters’ agreement, the drawings of 10 % of randomly chosen children were analyzed by both raters. 96 % of their answers to the questions about functions were identical, as well as 100 % of their evaluations of geometric shapes.

#### 2.1.4 Quantitative analysis

All drawings were scanned for digitalization, so that we could extract the exact location of each drawn curve in a cartesian plane and quantitatively estimate some parameters of the drawings. For non-oscillating functions, this digitalization allowed us to precisely extract the location of the first and last points of each drawing, which we used to quantify the curvature of exponential and quadratic patterns. If we qualitatively identified a curvature in the initial drawing, the more curved, the smaller the value of the x-position of the last point. For oscillating functions, this digitalization allowed us to extract the exact position of all extrema (minima and maxima) to precisely compute the period length and peak-to-peak amplitude of the pattern drawn by the child. Peak-to-peak amplitude increase, decrease or constancy was determined by calculating two separate linear regressions, one on local maxima (points whose y value is greater than its nearest neighbors) and one on local minima (points whose y value is smaller than its nearest neighbors). Peak-to-peak amplitude increase was thus characterized by a positive difference between the slopes of the two regressions; peak-to-peak amplitude stability by a difference close to zero; and peak-to-peak amplitude decrease by a negative difference.

### 2.2 Results

#### 2.2.1 Curve tracing as an implicit measure of fine motor skills

**Fig. 3:**
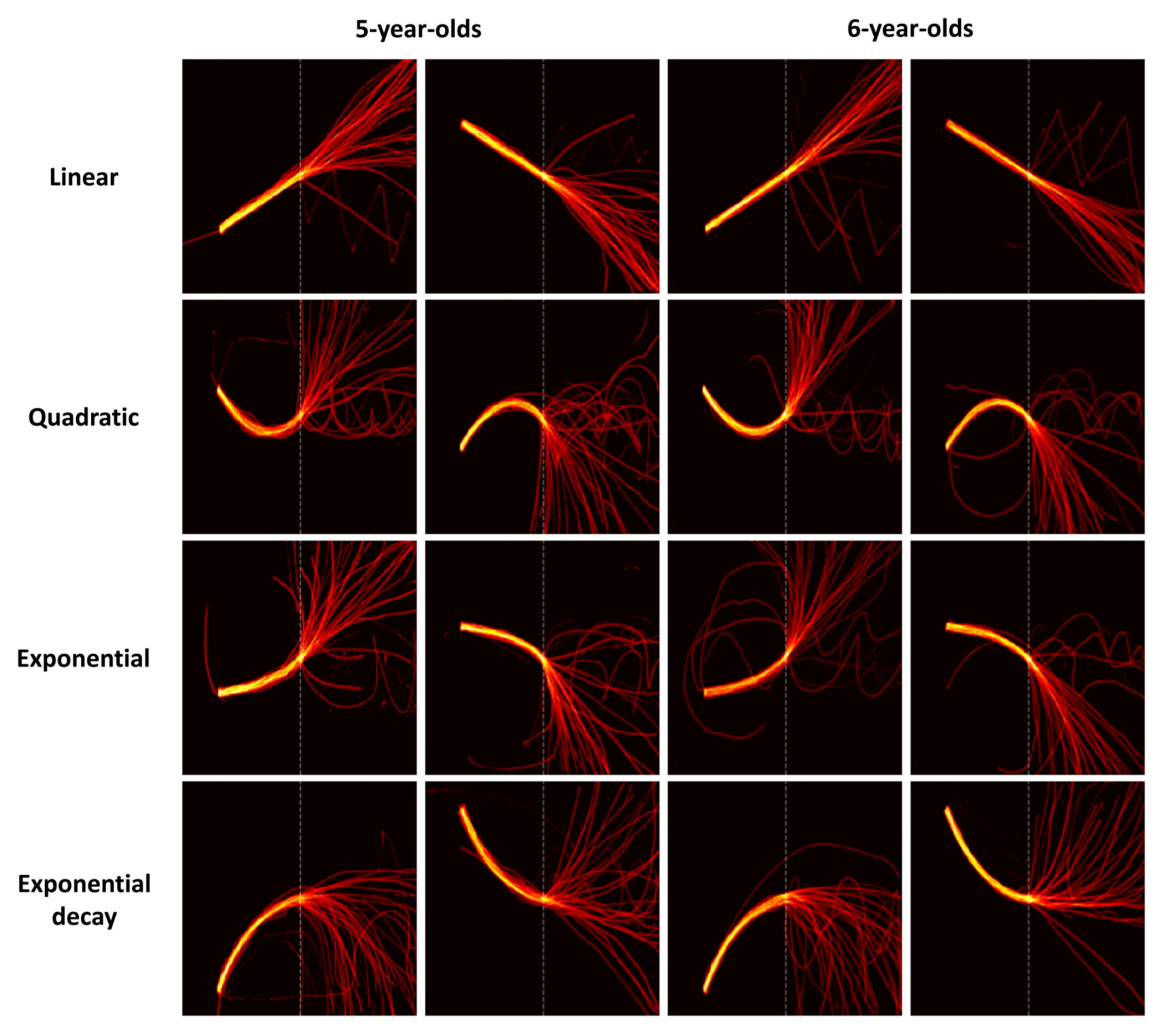
Prolongations of linear and non-linear functions. Each image presents all individual children’s drawings superposed, separately for the two age groups.

As outlined in the methods section, the children initially traced over the first portion of each drawing before prolonging it freely. This initial phase of pure retracing allowed us to indirectly evaluate the children’s fine motor skills. As presented in Fig. 3 (and in supplementary figs. 1, 3, and 5), which shows all digitalized responses, this phase was correctly executed by the totality of children. No child was thus excluded based on their ability to retrace the curve.

#### 2.2.2 Prolongations of non-oscillating functions respect their geometric properties

**Fig. 4:**
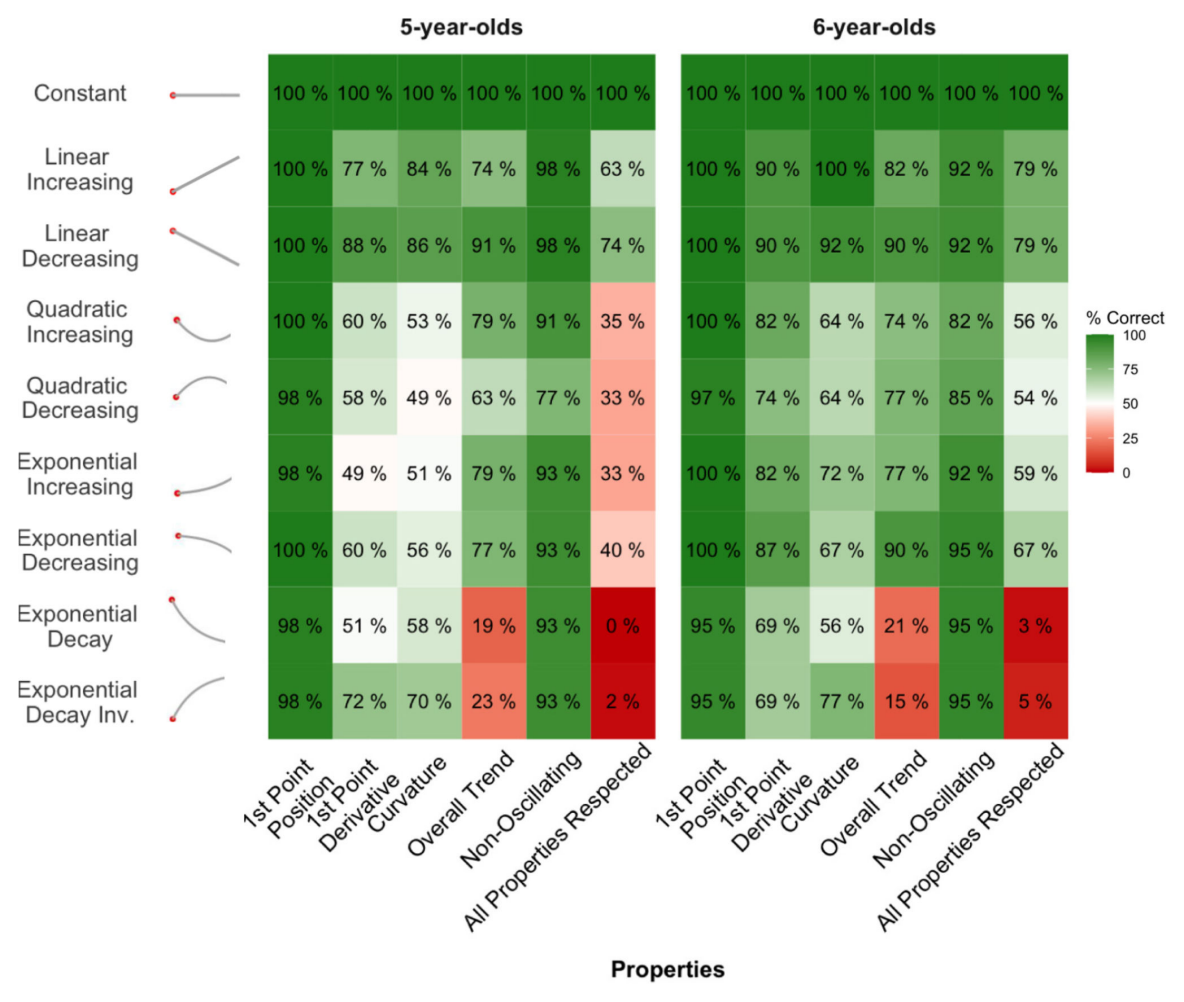
Percentage of responses respecting the properties of linear and non-linear functions. Each cell reports the percentage of drawings that respected a given functional property (x axis) for a given function (y axis), separately for the two age groups. The last column reports the percentage of drawings that respected all functional properties.

First, we looked at children’s responses for non-oscillating functions. As clear from Fig. 3, both kindergarteners (5-year-olds) and first graders (6-year-olds) were overall able to prolong the presented patterns as expected. To quantify these observations, we measured the percentage of correctly respected properties for each non-oscillating function (Fig. 4). First, the quasi totality of children respected the position of the first point of their prolongation (99 % of the kindergarteners’ trials, binomial test to evaluate if the proportion is different from 50 %: *p* <.001; and 99 % of the first graders’ trials: p <.001), meaning that they were able to adhere to the instruction of prolonging the pattern from where it stopped. Concerning the first point derivative, performance was above chance in kindergarteners and first graders (kindergarteners, 68 %, p <.001; vs. first graders, 83 %, *p* <.001), but improved with age (between-groups difference, χ^2^(1) = 18.99, p <.001), especially for non-linear functions (58 %, *p* <.01, vs. 77 %, p <.001; group-difference; χ^2^(1) = 18.96, p <.001).

Most participants also correctly prolonged these functions by drawing non-oscillating extensions (93 % of kindergarteners’ trials, p <.001; and 92 % of first graders’ trials, p <.001). The overall trend (i.e., whether the prolongation maintains the increasing, decreasing or constant trend of the initial pattern) was overall respected in both age groups (67 % of correct prolongations for 5-year-olds, p <.001, and 70 % for 6-year-olds, p <.001), with the notable exception of exponential decays, whose constant asymptotic trend on the right side of the drawing was correctly maintained only by a minority of children (19 % and 23 % for 5-year-olds; 21 % and 16 % for 6-year-olds).

Overall, 79 % of kindergarteners (p <.001), and 86 % of first graders (p <.001), respected all five parameters on linear functions. This percentage decreases slightly for non-linear non-oscillating functions, with only 24 % of kindergarteners and 41 % of first graders, who did so. The difference between linear and non-linear was significant for both kindergarteners (χ^2^(1) = 106.11, *p* <.001) and first graders (χ^2^(1) = 64.30, *p* <.001).

#### 2.2.3 Children understand monotonic prolongations but fail to distinguish between quadratics and exponentials

**Fig. 5:**
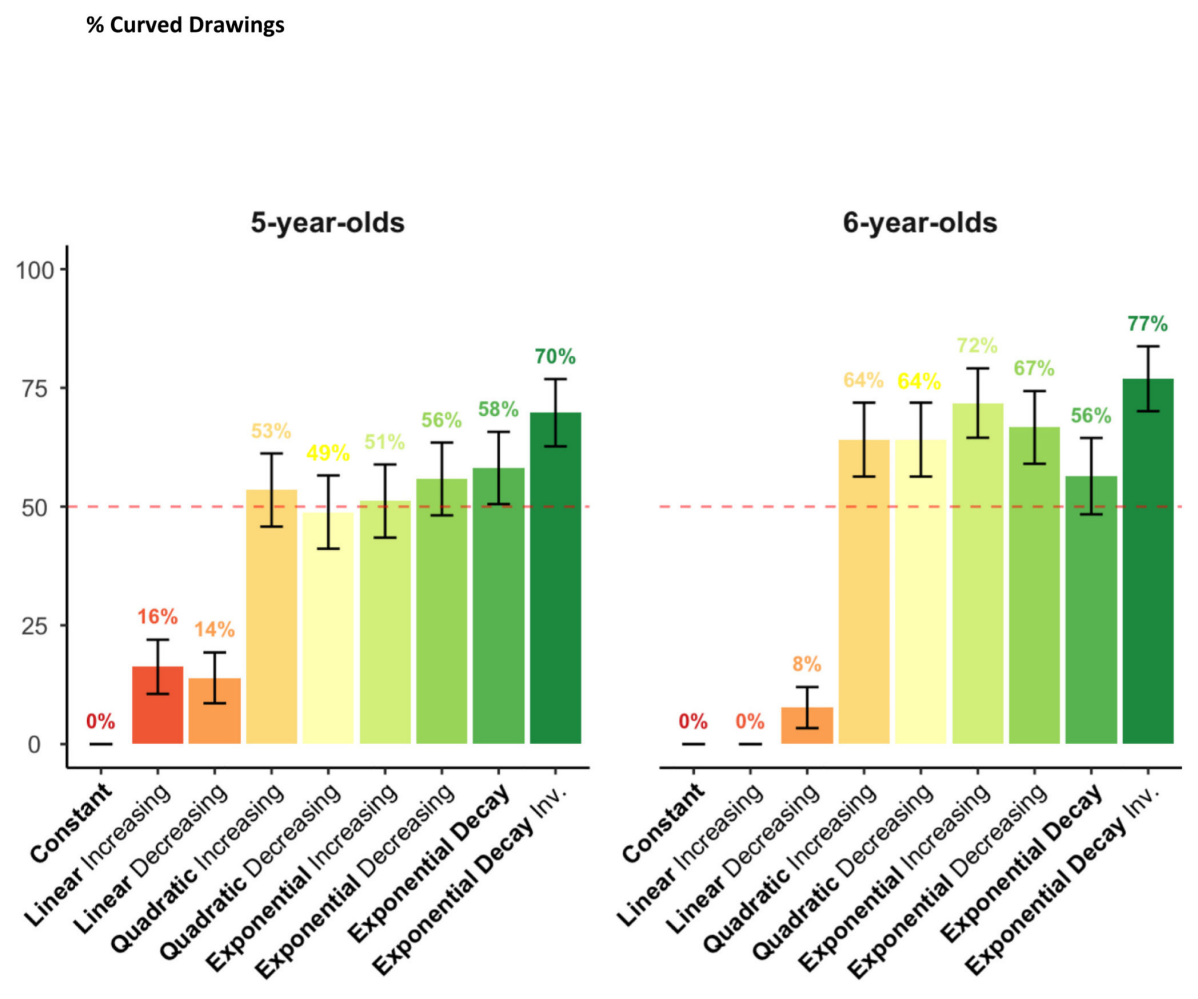
Percentage of curved prolongations for linear and non-linear functions. The percentage of patterns that were prolonged with a curve is presented as a function of pattern and age group.

To quantify the ability to distinguish linear from non-linear curves, we looked at the percentage of curved prolongations for both types of functions – i.e., whether the extended drawings were curved or straight (Fig. 5). Only on a minority of trials were linear functions erroneously prolonged with a curved pattern (7 % errors), although this happened significantly more often for kindergarteners than for first graders (10 % vs. 3 %; χ^2^(1) = 4.53, *p* =.033). Conversely, non-linear non-oscillating functions (quadratics and exponentials), for which a curved prolongation was expected, were correctly prolonged with a curved pattern on 56 % of trials for kindergarteners (*p* =.053), and 66 % of trials for first graders (*p* <.001). The difference between the two groups was significant (χ^2^(1) = 5.23, *p* =.022).

While children thus clearly differentiated linear and non-linear curves, we also wanted to know whether they represented exponential and quadratic patterns in distinct ways. To investigate this, we measured the x-position of the last point drawn by the children on the upper edge of the paper sheet (or bottom edge for decreasing functions). This point served as an indirect measure of the speed of growth of their drawing – the closer the point was to the top-right corner on this edge, the less curved the drawn pattern appeared. We observed that the difference between exponential and quadratic prolongations was never significant, either between increasing (5-year-olds: t(42) = −1.3, *p* =.21; 6-year-olds: t(38) =.42, *p* =.68) or decreasing patterns (5-year-olds: t(42) =.67, *p* =.51; 6-year-olds: t(38) = −2.0, *p* =.052).

#### 2.2.4 Children prolong oscillating curves with oscillating drawings

**Fig. 6:**
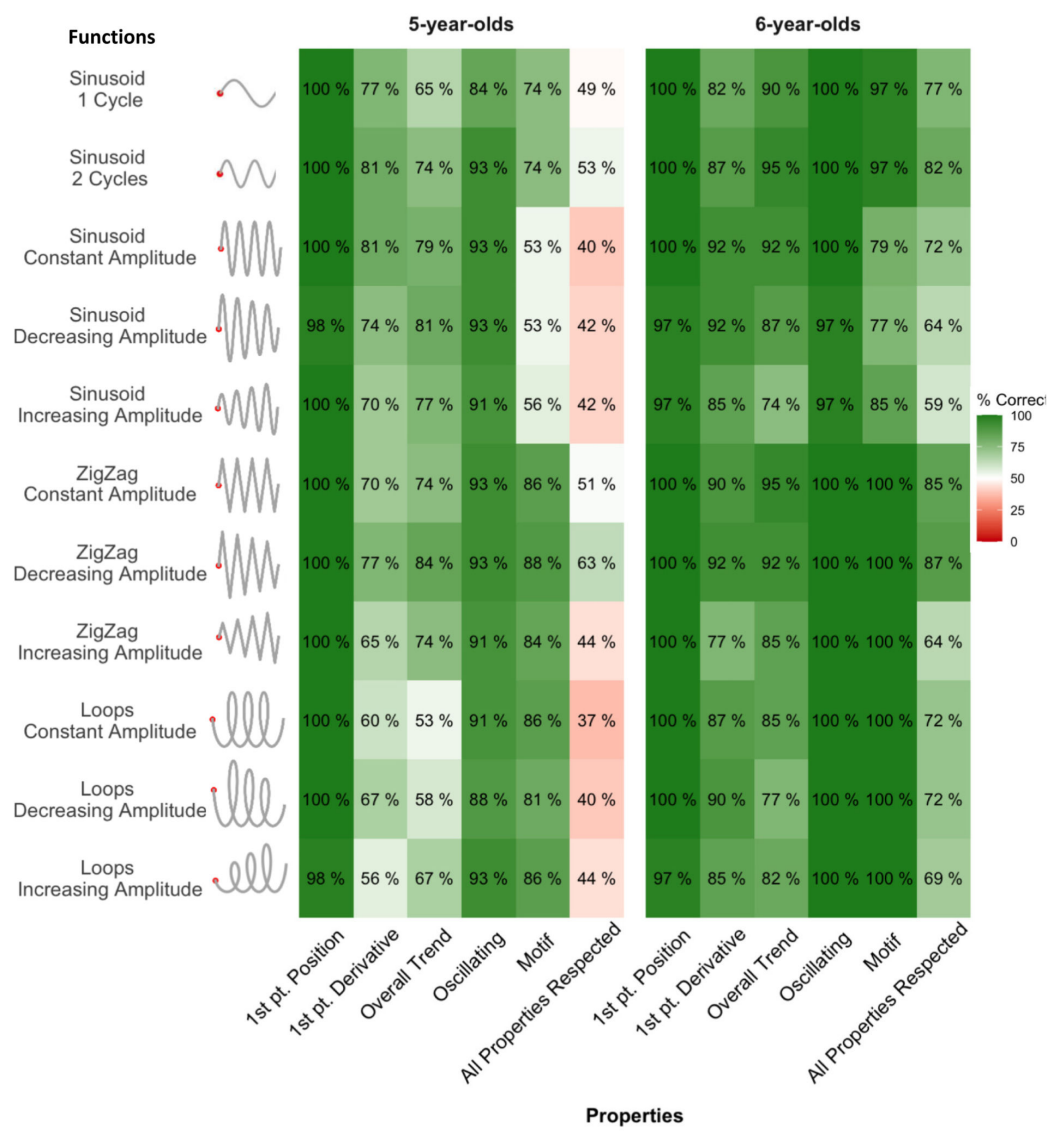
Percentage of responses respecting the properties of oscillating functions. Each cell reports the percentage of drawings that respected a given functional property (x axis) for a given function (y axis), separately for the two age groups. The last column reports the percentage of drawings that respected all functional properties.

Following the same qualitative approach described above, we measured the percentage of correctly respected properties in the drawings’ prolongations of oscillating functions (see Fig. 6 and supplementary fig. 1). As for non-oscillating functions, the first point position was respected for the quasi totality of drawings (99 % for kindergarteners, *p* <.001; and 99 % for first graders, p <.001; group difference, χ^2^(1) =.012, *p* =.91). The first point derivative and the overall trend were also respected by the majority of children in both age groups, but significantly more often for first graders (first point derivative: 71 %, *p* <.001, vs. 87 %, p <.001; group difference, χ^2^(1) = 34.82, p <.001; overall trend: 71 %, p <.001, vs. 86 %, p <.001; group difference, χ^2^(1) = 29.61, p <.001). Analogously, more than 9 out of 10 children correctly prolonged oscillating patterns with oscillating curves (again, significantly more so for first graders, 99.5 %, p <.001, vs. 91 %, p <.001; group difference, χ^2^(1) = 32.53, p <.001).

#### 2.2.5 Younger children often confuse sinusoids with zigzags

The main difference between the two groups was observed for the type of chosen motif (i.e., whether children correctly differentiate sinusoids, zigzags and loops). 6 year-olds were much more accurate than 5 year-olds at choosing the correct oscillating prolongation when presented with an oscillating pattern (74.6 %, p <.001, vs 94.1 %, p <.001; group difference, χ^2^(1) = 61.23, p <.001), especially for sinusoids (62 %, p <.001, vs 87 %, p <.001; group difference, χ^2^(1) = 31.7, p <.001). Interestingly, when children chose the wrong motif, they tended to produce a zigzag instead of a sinusoid. For first graders, 96 % (22 trials out of 23) of wrongly prolonged sinusoids were zigzags (p <.001). The opposite confusion never happened, as first graders always chose zigzag motifs when prolonging zigzag patterns. In kindergarteners, 82 % (50 trials out of 61) of wrongly prolonged sinusoids were zigzags (*p* <.001) and 75 % (6 trials out of 8) of wrongly prolonged zigzags were sinusoids (*p* =.14). In sum, while children distinguished oscillating and non-oscillating functions, they exhibited a bias towards drawing it with line segments, i.e., as a zigzag, although such errors decreased sharply from preschool to 1st grade.

#### 2.2.6 Prolongations of oscillating patterns respect the given periods

**Fig. 7:**
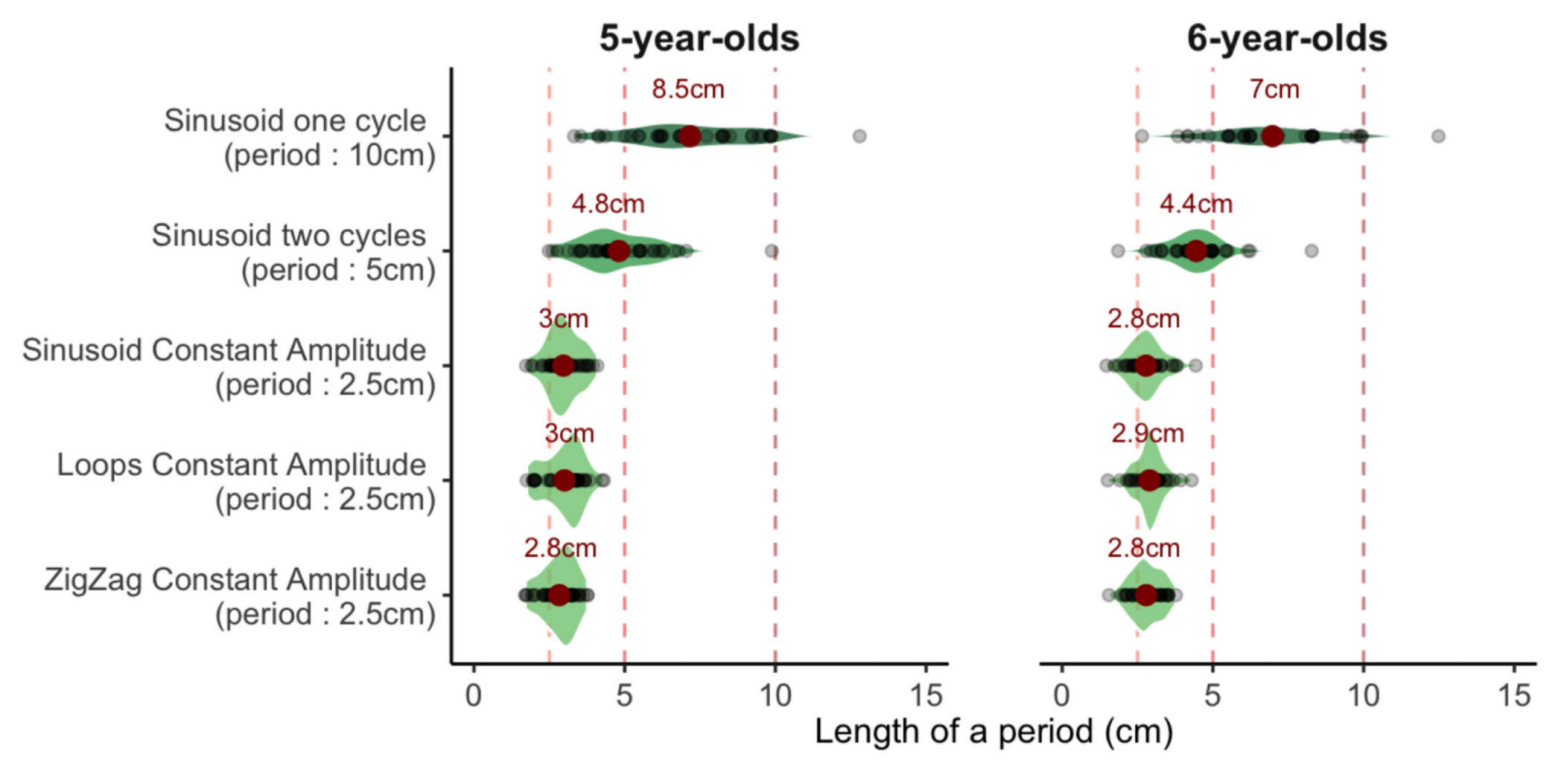
Respect of the period of oscillating patterns. For each oscillating pattern, the graph shows the distribution of period lengths in the children’s responses, separately for the two age groups. The red dot reports the average period. (For interpretation of the references to color in this figure legend, the reader is referred to the web version of this article.)

By digitizing the drawings, we conducted quantitative measurements, particularly on periods (1/frequency) drawn for the different oscillating functions. Fig. 7 shows the distribution of such periods. Participants in both age groups correctly drew a smaller period when the stimulus functions oscillated with a 2.5 cm period compared to those with a 5 cm period (5-year-olds: t(42) = 9.78, *p* <.001; 6-year-olds: t (38) = 11.29, p <.001), which in turn was smaller than that for periodic functions with a 10 cm period (5-year-olds: t(42) = −7.11, *p* <.001; 6-year-olds: t(38) = −8.95, *p* <.001).

#### 2.2.7 Children distinguish composite oscillating patterns with varying amplitude

**Fig. 8:**
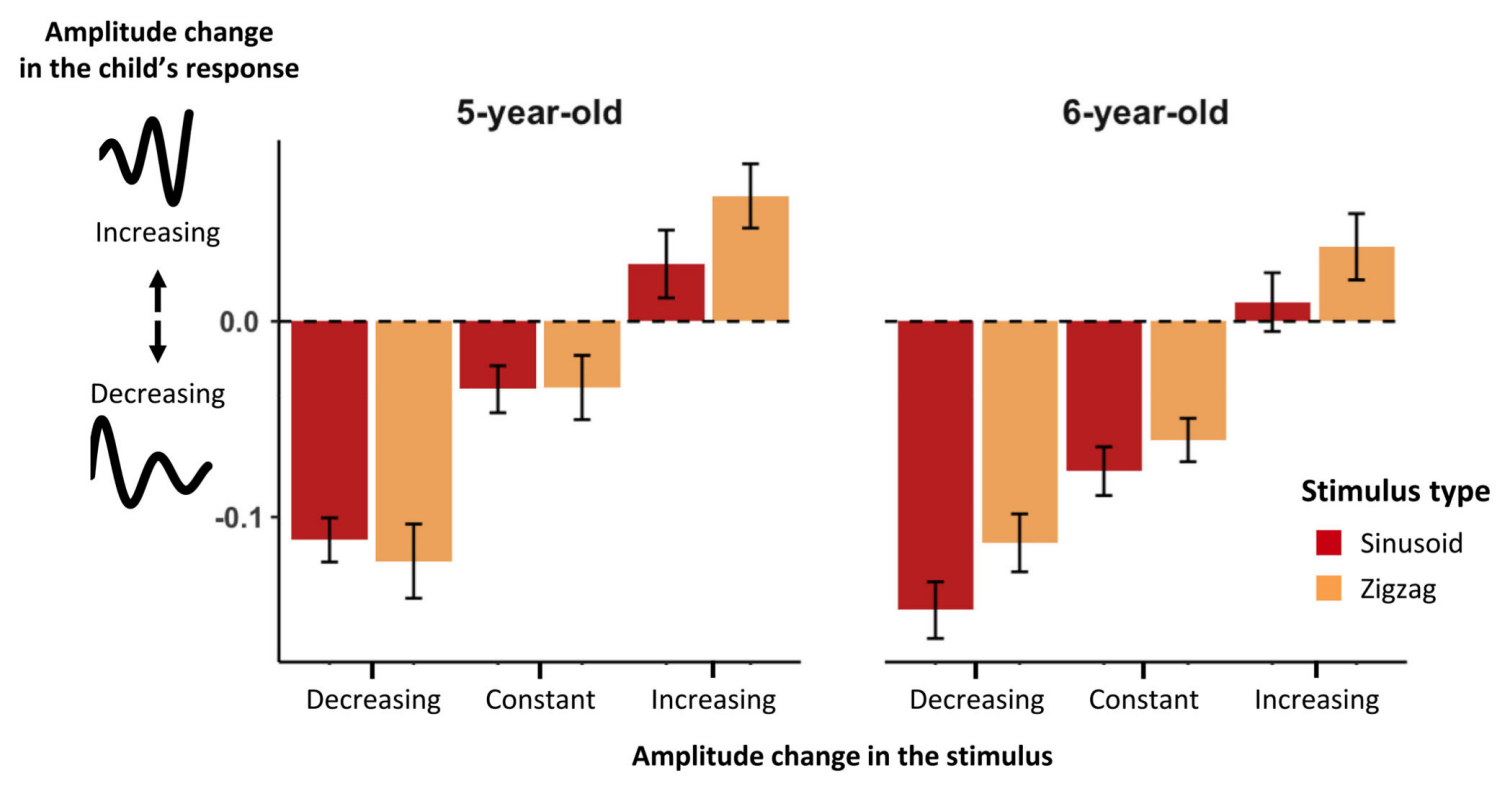
Respect of the amplitude change in oscillating patterns. The amplitude change in a child’s drawing (difference in slope of the lines through the maxima and through the minima) is plotted for each pattern on the x axis and for each age group. A positive change means the drawn pattern increased in amplitude, a negative change means it decreased, a change close to zero means it remained constant.

To investigate children’s understanding of compositionality, we included composite patterns such as sinusoids and zigzags whose peak-to-peak amplitude could remain constant, increase or decrease over their periodic cycles. To explore whether children respect the evolution of these peak-to-peak amplitudes, as explained in the methods’ section, the difference between the slope of the regression lines for maxima and minima was used to characterize whether the child’s response exhibited an increasing, constant or decreasing oscillation amplitude (Fig. 8). A positive difference means an increase in peak-to-peak amplitude, whereas a negative difference means a decrease in peak-to-peak amplitude. As many children drew loops with little precision, we report here only the results for sinusoids, zigzags, and stairs (discussed in the next section). We also excluded from the analysis all drawings whose prolongation was not oscillating (4.5 % of the drawings).

We observed that when the initial pattern had a constant peak-to-peak amplitude, children in both age groups tended to draw functions of slightly decreasing peak-to-peak amplitude (5-year-olds: sinusoid – t(39) = −2.90, *p* <.01; zigzag - t(39) = −2.06, *p* <.05; 6-year-olds: sinusoid - t(38) = −6.15, p <.001; zigzag - t(38) = −5.47, p <.001). Crucially, when the initial pattern showed a decreasing peak-to-peak amplitude, the trend for a decreasing amplitude was larger (5-year-olds: sinusoid - t(39) = −5.85, p <.001; zigzag - t(39) = −4.97, *p* <.001; 6-year-olds: sinusoid - t(37) = −5.48, *p* <.001; zigzag - t(38) = −4.45, p <.001). The opposite trend was observed for patterns of increasing amplitude. Children in both age groups tended to prolong them with an increasing amplitude, although this tendency, relative to zero, was only significant for zigzags (5-year-olds: sinusoid - t(38) = 1.69, *p*. = 099; zigzag - t(38) = 3.90, p <.001; 6-year-olds: sinusoid – t(37). = 65, *p* =.52; zigzag - t(38) = 2.25, p <.05). Overall, when the initial pattern showed an increasing peak-to-peak amplitude, the change in amplitude drawn by children was larger than for patterns with constant amplitude (5-year-olds: sinusoid - t(38) = −3.08, p <.01; zigzag - t(38) = −5.34, p <.001; 6-year-olds: sinusoid - t(37) = −5.20, p <.001; zigzag - t(38) = −6.00, *p* <.001). Taken together, these results strongly suggest that children’s changes in peak-to-peak amplitude reflected a correct understanding of the stimulus pattern and its amplitude change.

#### 2.2.8 Interindividual differences and progression in the drawing of oscillating composite patterns

As described earlier, most children seem to respect the oscillatory nature of sinusoids and zigzags, others correctly follow the exact motif, and fewer grasp the composite nature of some of the functions. But do these differences reflect a consistent progression from one stage to the next across children? To investigate this aspect, we assessed them within each child. Compliance with the composite aspect was determined by applying a threshold to changes in amplitude: patterns with increasing amplitude were considered correct if the change exceeded 0.05; those with decreasing amplitude were considered correct if the change was below −0.05; and those with constant amplitude were considered correct if the change fell between −0.05 and 0.05. A large proportion of children respected all three properties (40 % in 5-year-olds and 44 % in 6-year-olds), while very few children respected none (8 % in 5-year-olds and 1 % in 6-year-olds). More interestingly, among children who did not adhere to at least one of these properties, compositionality (amplitude change) was more frequently neglected (30 % in 5-year-olds and 47 % in 6-year-olds) than motif (12 % in 5-year-olds and 4 % in 6-year-olds; see Supplementary Fig. 2). Crucially, 77 % of kindergarteners who respected the compositional nature of the pattern also chose the correct motif (and this percentage increased to 93 % for first graders). These observations suggest three successive stages in children’s drawings of oscillating composite patterns: 1) recognizing the oscillating nature of the pattern; 2) choosing the correct motif (zigzag or sinusoid); and 3) respecting its compositional nature, i.e., the direction of its amplitude change.

#### 2.2.9 Stair patterns are difficult for young children and their change in amplitude is not always respected

**Fig. 9:**
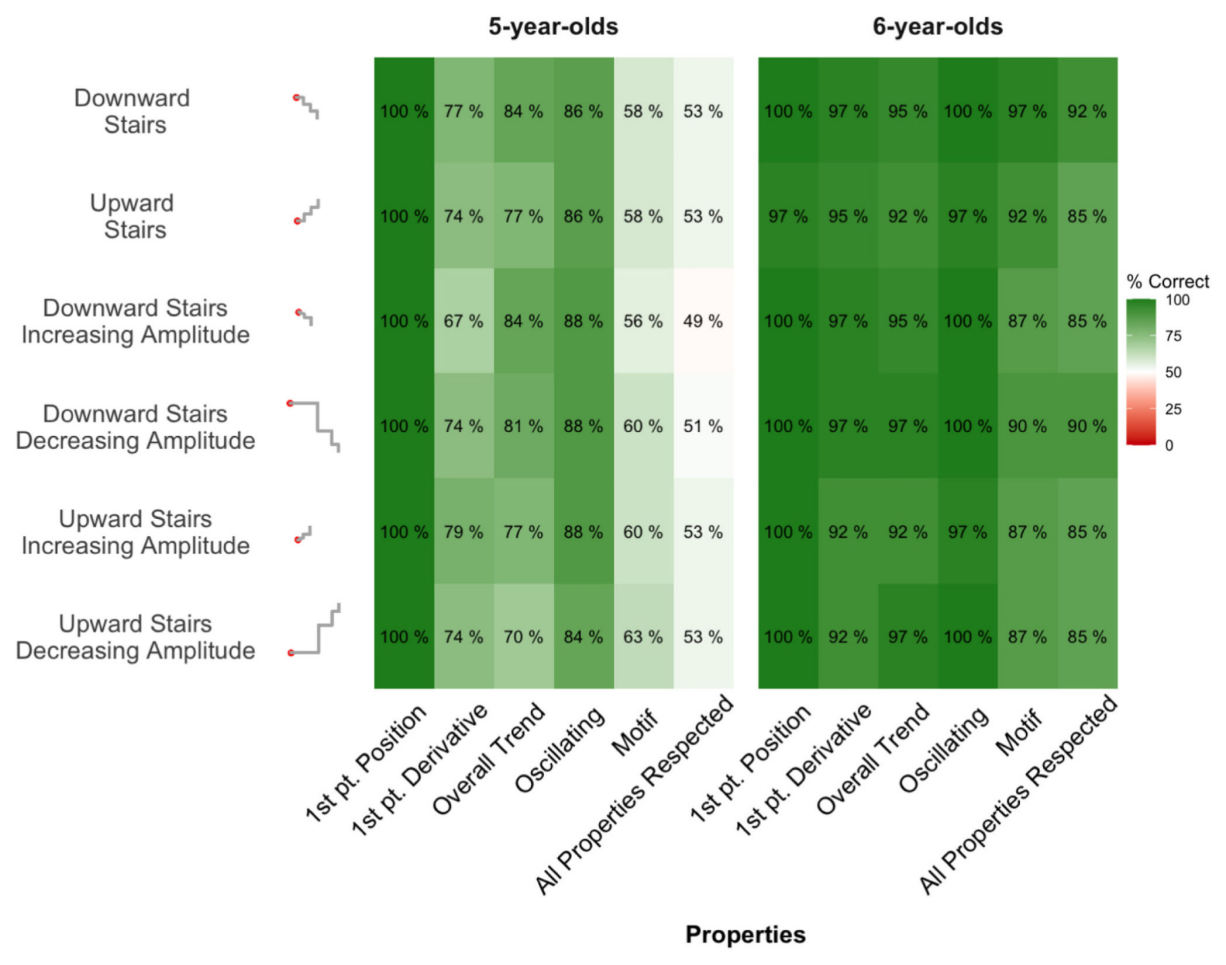
Respect of the properties of staircases. Each case reports the percentage of drawings that respected a given functional property (x axis) for a given function (y axis), separately for the two age groups. The last column reports the percentage of drawings that respected all functional properties.

Staircase stimuli were included in order to investigate the understanding of patterns that varied both in amplitude and frequency. The results from the qualitative analysis are presented in Fig. 9 (supplementary fig. 3 shows all digitalized prolongations). As for other periodic functions, almost all children were able to correctly place the first point (100 % of kindergarteners’ trials, *p* <.001, and 99.5 % of first graders’ trials, p <.001). They had more trouble identifying the correct line orientation (derivative) at first point, and even more so kindergarteners than first graders (74 %, *p* <.001, vs. 95 %, *p* <.001; group difference, χ^2^(1) = 38.96, p <.001). The overall trend (upward or downward) was correctly identified by a vast majority of children, with a significant improvement in performance between kindergarteners and first graders (79 %, p <.001, vs. 95 %, p <.001; group difference, χ^2^(1) = 25.98, p <.001). Finally, a vast majority of kindergarteners and even more first graders correctly prolonged staircase patterns with oscillating curves (87 %, p <.001, vs. 99 %, p <.001; group difference, χ^2^(1) = 25.69, p <.001); but first graders more often chose the correct oscillating pattern, i. e., a stair-like pattern (59 %, *p* =.49 vs. 90 %, p <.001; group difference, χ^2^(1) = 59.15, p <.001).

To study children’s changes in amplitude and frequency, we used the same method as described for the other oscillating patterns: we identified the position of the extrema and plotted regressions on the minima and on the maxima separately. We then computed the slope difference. We removed from this analysis drawings that were not oscillating (7.3 % of the drawings) and drawings whose oscillating pattern was not a staircase, a zigzag or a sinusoid (7.5 % of the remaining drawings). Results are depicted in supplementary fig. 4. Statistical analyses revealed that patterns with constant and increasing amplitude were all prolonged with a constant amplitude in both kindergarteners and first graders (*p* >.41 for all *t*-tests) except downward stairs with increasing staircases in first graders, which was prolonged with an increasing amplitude (t(36) = 3.07, *p* <.01). Conversely, a decrease in amplitude was respected except for upward stairs with decreasing amplitude in first graders, which were prolonged with a constant amplitude instead (5-year-olds: upward stairs – t(33) = −3.35, p <.01; downward stairs - t(32) = −2.47, *p* <.05; 6-year-olds: upward stairs - t(37) = −.063, *p* =.95; downward stairs - t(37) = −2.43, p <.05).

#### 2.2.10 Children correctly complete geometric shapes

As explained in the methods’ section, at the end of the experiment, children were asked to complete two spirals (one spiraling towards the inside, one spiraling towards the outside), a circle and a square. Despite the instructions did not change for these stimuli (they were still invited to prolong the “path”, and the use of this term might have not elicited the production of a closed figure; Lin & Dillon, 2023), most participants in both age groups succeeded in the task (supplementary fig. 5 and 6), with an average performance of 84 % correct (p <.001) for kinder-garteners, and of 85 % correct (p <.001) for first graders.

## 3 Experiment 2

### 3.1 Methods

#### 3.1.1 Participants

We tested 48 kindergarteners (26 girls, average age: 60 months) and 28 first graders (19 girls, average age: 73 months), from 4 different schools. As in experiment 1, parents were informed of the purpose of the study and the possibility for them to object to their child’s participation via a form, given to the child by the teachers. Participants were tested by our research team during school time, in a quiet room close to the classroom.

#### 3.1.2 Experimental procedure

The design of the task and stimuli are presented in Figs. S7 to S10 in supplementary materials. The task followed a multiple-choice paradigm where children were asked to select the correct prolongation of a mathematical graph among six options. Each trial began with a 1-s red fixation point, followed by a 2-s presentation of a 2 × 3 grid displaying the same first half of a graph six times. The test phase followed for up to 15 s, during which the initial graph halves were presented alongside six possible prolongations – one correct and five violating key mathematical properties (e.g., continuity, curvature). Children were instructed to “choose the extension you feel is the best continuation of the path you see at the beginning”.

A training phase preceded the main task, consisting of four trials with animal bodies and two with geometrical shapes. The animal body or the shape could be prolonged either correctly or according to one out of five distortions (translation, rotation, color change, enlargement or reduction). Each response during training received audio feedback. The main task comprised three blocks of 22 trials each, presented in a fully randomized order. Each block included 19 trials with mathematical functions and three easier trials from the training set. Stimuli fell into three groups: 7 *non-oscillating functions*, 2 *oscillating functions*, and 10 *oscillating functions with varying amplitude or frequency*.

*Non-oscillating* functions correspond exactly to those used in Experiment 1, excluding the exponential decay function. Incorrect continuations violated one of five properties: 1) continuity at the first point (introducing a constant shift), 2) derivative at first point (altering the derivative at the transition point), 3) overall trend (opposite variation direction), 4) curvature (linear continuation for non-linear functions and vice-versa), 5) non-periodicity (continuation with a sinusoidal function). A similar approach was used for *oscillating functions*, which included sinusoid and zigzag patterns, both with identical constant frequency and amplitude. The same five violation principles applied, except that the curvature violations were replaced by motif violations (i. e., extending a sine wave with a zigzag pattern and vice-versa). Each incorrect continuation violated only one properly (except when mathematically infeasible).

Finally, a set of stimuli was designed to investigate compositionality, using five generative functions: three from Experiment 1 (a sinusoidal function with constant frequency and amplitude, one with increasing amplitude, and one with decreasing amplitude) and two new functions (sinusoidal functions with the same amplitude but quadratically increasing or decreasing frequency). The five incorrect continuations included a violation of: 1) motif; 2) amplitude (i.e., smaller or larger); 3) amplitude trend (i.e., amplitude increasing or decreasing); 4) frequency (i.e., smaller or larger); 5) frequency trend (i.e., frequency increasing or decreasing).

#### 3.1.3 Data analysis

We removed from the analysis participants who responded randomly (*p* >.05 for a binomial test with a chance level of 1/6 – 3 kindergarteners and 3 first graders) and participants whose accuracy on training trials was less than 70 %, considering that those children did not properly understand the task (3 kindergarteners and 1 first grader). For the remaining 66 children, we removed trials for which the response time was faster than 1 s (1.9 % of the trials). We reasoned that such fast responses could only reflect arbitrary choices or finger slips, as they did not reasonably allow children to perceive and analyze all six options. Performance across experimental conditions was then compared using chi squared test and performance for a specific experimental condition was compared to chance level using a binomial test with a chance level of 1/6.

### 3.2 Results

#### 3.2.1 Children recognize the correct prolongation of functions in a multiple-choice task

In the non-drawing version of the experiment, participants first saw half of a mathematical graph (hereafter called the sample) and then chose the correct prolongation among six options, five of which violating one mathematical property (supplementary fig. 7). Findings confirmed the results obtained in the drawing task: children of both ages chose the correct prolongation of the sample significantly more often than chance for all non-oscillating (supplementary fig. 8) and oscillating (supplementary fig. 9) functions (*p*-values <.05 for all binomial tests, 65 % of correct responses in both kindergarteners and first graders). They also selected the correct answer more often than any of the aberrant choices (prolongations violating the continuity at first point, the derivability at first point or the periodicity – *p* <.05 for all chi squared tests).

#### 3.2.2 Distinctions between linear and non-linear functions

Participants often misunderstood the curvature of exponentials, selecting a linear continuation almost as frequently as the correct exponential one (e.g., for exponential increasing: 49 % vs 42 % for 5-y-olds; difference: χ^2^(1) = 1.02, *p* =.31; 54 % vs 43 % for 6-y-olds; difference: χ^2^(1) = 1.36, *p* =.24). In contrast, they recognized the curvature of quadratic functions, choosing the correct continuation significantly more often than the linear alternative, though errors still occurred (e.g., for quadratic increasing: 60 % vs 26 % for 5-y-olds; difference: χ^2^(1) = 7.00, *p* <.001; and 53 % vs 26 % for 6-y-olds, difference: χ^2^(1) = 3.14, p <.001).

#### 3.2.3 Confusions between sinusoids and zigzags as in the drawing task

Results on oscillating functions replicated the confusion between zigzags and sinusoids observed in the drawings. When selecting the wrong continuation on sinusoids, 6-year-olds selected the zigzag prolongation significantly more often than any others (*p* <.05 for all chi squared tests). A similar trend was observed in 5-year-olds (p <.05 for all chi squared tests except for the comparison with the distractor violating the trend, which was not significant: χ^2^(1) = 0.92, *p* =.34). Conversely, when selecting the wrong continuation for zigzags, 5-yearolds selected the sinusoid prolongation significantly more often than any others (p <.05 for all chi squared tests), with a similar trend observed in 6-year-olds, though not always significant (p <.05 for all chi squared tests except for the comparison with the distractor violating the trend, χ^2^(1) = 0.21, *p* =.65, and the comparison with the distractor violating the periodicity, χ^2^(1) = 0.21, p =.65).

#### 3.2.4 Simplicity bias for composite functions

For composite functions with varying amplitude and frequency (supplementary fig. 10), just as when drawing, children made many more mistakes. However, regardless of the variations of frequency and amplitude in the stimulus, children predominantly chose the prolongation with constant amplitude and frequency. They did so for the sinusoid with constant amplitude and frequency, where it was the correct answer (p-values <.05 for all chi squared tests), but also for the sinusoid with decreasing amplitude, where it was not the correct answer (p-values <.05 for all chi squared tests). For the sinusoid with increasing amplitude, 5-year-olds selected equally often the correct answer and the prolongation with constant amplitude and frequency (χ^2^(1) = 1.23, *p* =.27), while 6-year-olds favored the correct answer, the prolongation with constant amplitude and frequency, or the zigzag prolongation (*p* >.05 for all chi squared tests). Similarly, for sinusoid with increasing frequency, 5-year-olds chose equally often the correct answer and the prolongation with constant amplitude and frequency (χ^2^(1) = 2.12, *p* =.14), whereas 6-year-olds predominantly selected the latter (p <.05 for all chi squared tests). Finally, for the sinusoid with decreasing frequency, both 5-year-olds and 6-year-olds most often selected either the prolongation with constant amplitude and frequency or the zigzag prolongation (5-year-olds: χ^2^(1) = 0.61, *p* =.44; 6-year-olds: χ^2^(1) = 1.06, *p* =.30).

In spite of those errors, direct comparisons of the choice of a given target as a function of the sample oscillating curve showed that children did not fully confuse the curves with variable frequency, variable amplitude, or both parameters constant. The “variable frequency” target was chosen significantly more often when the sample varied in frequency than when it did not (5-year-olds: 21 % vs. 7.6 %, χ^2^(1) = 44.38, *p* <.001; 6-year-olds: 17 % vs. 9.6 %, χ^2^(1) = 7.92, *p* =.0049). Likewise, the “variable amplitude” target was chosen more often when the sample varied in amplitude than when it did not (5-year-olds: 20 % vs. 12 %, χ^2^(1) = 10.17, *p* =.0014; 6-year-olds: 24 % vs. 8.9 %, χ^2^(1) = 21.6, p <.001). And finally, the “constant sinusoid” target was chosen more often when the sample was constant than when it did not, except in 5-year-old, where the difference did not reach significance (5-year-olds: 36 % vs. 41 %, χ^2^(1) = 2.32, *p* =.13; 6-year-olds: 35 % vs. 47 % χ^2^(1) = 6.73, *p* =.0095). Thus, the main problem that children had was to distinguish these patterns from each other, particularly the sinusoid and the zigzag, which could be attributed to the small size of the differences on screen.

## 4 Discussion

### 4.1 Drawing as a method to study patterns’ understanding

In our first experiment we asked children to freely draw the extension of a given visual pattern, created from simple mathematical functions (e.g., linear, quadratic, exponential, sinusoid) and their compositions. This original method allowed us to quantitively investigate children’s implicit understanding of concepts such as linearity or curvature, which would be much harder to study using classic tools such as, for example, building blocks. Notably, asking children to freely draw the prolongation of functions allows to investigate their mental representations of visual patterns without constraining the set of possible responses, thus leaving aside any preconceived ideas of how children would behave.

First, we successfully demonstrate that both kindergarteners and first graders could tackle this task. Indeed, they were able to prolong both linear and non-linear functions, generally respecting properties such as first point location and derivative, curvature, and overall trend. Children accurately distinguished linear from non-linear functions, drawing quadratic and exponential functions with a curvature that was not present when prolonging linear functions. Additionally, they were also able to grasp the periodic nature of oscillating motifs, including sinusoids and zigzags. These findings are in line with studies showing that 5-year-old children distinguish linear from non-linear increases in the number of discrete items in a container (Ebersbach, Van Dooren, Goudriaan, & Verschaffel, 2010) and that from the age of 4, children are also able to distinguish visual items whose total area increases linearly or non-linearly (Coates et al., 2023). Children’s drawings in our study clearly confirm that they had no trouble prolonging non-linear or oscillating functions, thus providing evidence that children’s understanding of patterns is not limited to simple ones, such as repeating and alternating motifs or patterns growing by one item at the time (Wijns, Verschaffel, De Smedt, & Torbeyns, 2021). On the contrary, a rich catalogue of functions seems available early on in development, perhaps as rich as the one already observed in human adults in extrapolation tasks (Ciccione et al., 2022; Ciccione & Dehaene, 2021; Schulz et al., 2017).

Our study also suggests that children did not distinguish non-linear functions at a finer detail, as they did not differently prolong exponentials and quadratics. However, it is worth noting that the expected correct prolongations for these two functions were rather similar to each other. Future studies should present children with more distinct types of non-linear functions, whose correct prolongations differ more from each other, in order to better test children’s implicit and intuitive understanding of concepts such as asymptote and acceleration.

### 4.2 Ruling out the motor hypothesis

Because drawing tasks rely heavily on motor skills, some errors observed in children’s drawings – and some age-related improvements – could be attributed to motor constraints rather than conceptual understanding or progress. Conversely, some could argue that success in children’s drawings might only reflect motor entrainment rather than conceptual understanding: children may only prolong patterns by continuing the same motor program initiated during the curve-retracing phase. To test whether children’s behaviors in the drawing task was merely a result of motor constraints or motor entrainment, we designed a second task in which the motor component was completely removed. By presenting the same stimuli, but now in a multiple-choice paradigm, we asked a different sample of children to select the correct continuation of each function among several alternatives – a multiple-choice approach similar to that previously used with geometric shapes (Dehaene, Izard, Pica, & Spelke, 2006; Izard & Spelke, 2009; Sablé-Meyer et al., 2021, 2022). The findings from this task closely mirrored those of the drawing experiment. For example, for most presented curves, children of both ages significantly preferred a prolongation that followed the correct mathematical function used to draw the initial curve than prolongations violating some properties of the initial curve. They also frequently confused sinusoids for zigzags and vice versa (as when drawing) and misunderstood the curvature for non-linear functions, sometimes favoring a linear continuation when a curved one was expected. Oscillating patterns, particularly composite ones, led to a lesser performance, yet still influenced by all the parameters of the presented curve, including frequency and amplitude variations.

Taken together, these findings suggest that children’s good performance in the drawing task is not merely a byproduct of motor entrainment, as their behavior is similar in the multiple-choice task. Likewise, their errors in drawings cannot be solely attributed to motor limitations, since the same pattern of mistakes appeared without the drawing component. This suggests that children’s understanding of mathematical functions, as revealed through drawing, is primarily driven by a conceptual representation, with motor factors playing only a minor role.

### 4.3 Conceptual versus perceptual saliency

In both experiments, children – particularly kindergarteners – struggled to differentiate certain functions: notably the graphs of sinusoidal and zigzag functions, as well as the graphs of exponential and quadratic functions. Several reasons could underlie these confusions. They may be *conceptual*, as we postulate, wherein certain functions (e.g., sinusoids and zigzags) are grouped under the same abstract category. In other words, we could speculate that these confusions might reveal children’s tendency to detach from local non-salient aspects of the pattern (such as the difference between an angular point in a zigzag and a curve in a sinusoid), and to focus on global structure (i.e., the periodic nature of the motif). While a *motor* explanation can be ruled out based on the results of the multiple-choice task (as described in the previous section), a *perceptual* account remains plausible: these functions may be too visually similar to be correctly distinguished. However, it seems unlikely that a 5-year-old would be unable to visually discriminate between a sinusoid and a zigzag while a 6-year-old would suddenly acquire this ability. A conceptual/attentional account seems more likely, with older children jointly attending to both local and global features of the display while younger ones would focus solely on global structure. Future studies are needed to directly test the perceptual discriminability of these functions, for instance by using larger and more salient visual stimuli, and to assess the relative contributions of visual discrimination, attention and conceptual development at this age.

### 4.4 The development of pattern comprehension

An improvement in performance was seen from kindergarteners to first graders. Older children more frequently respected the first point derivative, for both oscillating and non-oscillating functions, and better distinguished linear from non-linear functions – curving non-linear functions more often, and linear ones less so. Of greater interest, first graders also more often chose the correct oscillating pattern when prolonging zigzags, sinusoids or loops – and, as just discussed, while kindergarteners grasped the general concept of oscillation, first graders showed a finer ability to distinguish between different types of oscillating functions (zigzags from sinusoids).

These findings align with prior evidence that pattern comprehension evolves during development (Wijns, Torbeyns, De Smedt, & Verschaffel, 2019), and our study suggests that while many different functions are grasped by 5 year-olds, their understanding gets refined with age. Indeed, in the limited age-range that we tested, not all patterns showed age-related improvements. For example, as already mentioned, children of both age groups did not differently prolong exponentials and quadratics, thus providing no evidence that they understand the difference. The correct prolongations for these two functions were, however, only subtly different, and future studies should introduce more distinct non-linear patterns to better assess children’s intuitive understanding of advanced concepts such as derivative, acceleration and asymptote.

Our drawing task shares several characteristics with “complete the sequence” tests used to measure fluid intelligence (Kent, 2017). Most notably, it proposes open-ended questions, meaning there is no absolute correct response other than what the experimenter has in mind. In this sense, the increase in performance with age could be explained by the fact that first-graders understood more precisely what the instructors had in mind, as well as by a genuine improvement in protomathematical intuitions. Whether intuitions of graphic patterns are just the expression of a general fluid intelligence factor or involve domain-specific geometric knowledge, perhaps specifically linked to mathematical abilities by means of a shared language of thought, should be further investigated, especially in very young children. Indeed, while Giofrè, Mammarella, and Cornoldi (2014), in 4th and 5th graders, showed that intuitive geometry was correlated with fluid intelligence and did not provide a unique predictor of academic math achievement, Zippert, Clayback, and Rittle-Johnson (2019) found that, in preschoolers, pattern comprehension predicted math knowledge over and above the impact of fluid reasoning.

### 4.5 Early intuition versus explicit learning

The word “intuition” has multiple meanings. In this manuscript, we use the term “intuitive” in the same sense as in intuitive physics or intuitive psychology, as knowledge that appears immediate and self-evident (Fischbein, 1982), without complex deliberation in working memory (Evans & Stanovich, 2013), and in the absence of accumulated training or explicit instructions, or even in spite of it (McCloskey, Washburn, & Felch, 1983). Although our children might have been familiar with some of the patterns used, they did not have to be trained to perform any of our tasks, nor did they receive feedback on their performance. More in detail, our tasks required judgments that were both immediate (since children were invited to answer spontaneously, without complex thinking) and self-evident (since they were not asked to provide any justification or formal demonstration to their answers), two aspects commonly associated to intuitions (Fischbein, 1982). Our findings show that kindergartners and first graders could extrapolate the natural continuation of a line, or identify its correct prolongation, without relying on explicit strategies, mathematical calculations, or working memory manipulations, whose use is often considered a signature of deliberate non-intuitive processes (Evans & Stanovich, 2013): in fact, unlike other tasks described in the literature (which oblige participants to keep in mind and manipulate a series of items before providing a response), our tasks maintained the main information (i.e., the generative function), always available to the child, thus avoiding the need to memorize it before answering. It is also worth noting that “intuitive” here does not necessarily mean “fast” or “automatic”: as previously been shown, intuitive responses can still be slower than more reflexive ones (see e.g., Evans, Dillon, & Rand, 2015). Lastly, these intuitions are likely to be strongly rooted in evolutionarily ancient systems. In this respect, we speculate that intuitions of space might be similar to intuitions of quantities (the so-called approximate number system), that psychophysically vary as a function of the Weber law, the signature of an evolutionarily ancient “number sense” (Dehaene, 2009).

One might argue that children’s success in the drawing task was not the expression of an intuitive and implicit understanding of visual functions but rather the result of training with similar tasks in a school environment. However, while we did take advantage of the fact that these activities are already present and appreciated by both children and teachers, the functions we proposed to our participants went far beyond the ones presented at school. Indeed, while the patterns in children’s school activities may include periodic ones such as sinusoids and zigzags, they certainly do not include exponentials, quadratics, or composite periodic patterns. This means that, when facing such peculiar functions, children had no other choice than answering on the grounds of their intuitions, since they could not resort to any formal knowledge about the mathematical behavior of those functions. Moreover, our second task, which required selecting continuations without drawing, was totally novel in a school context. Ideally, future studies should investigate the possible role of training by testing children with no formal school experience, such as those living in remote societies (as recently done in the study of graphics’ perception; Ciccione et al., 2023). It seems likely that explicit training with patterns (and mathematics in general) would refine the implicit and intuitive understanding of functional relationships, similarly to what happens to the refinement of the intuitive number sense with education (Dehaene, 2011; Dehaene, Izard, Spelke, & Pica, 2008; Siegler & Opfer, 2003). Vice-versa, training children’s proto-mathematical intuitions about functional relationships might result in later advantages in formal mathematics, an hypothesis that has already received some support (Wijns et al., 2019).

### 4.6 Drawing as a window to the study of compositionality

Our drawing study reveals that both kindergarteners and first graders, without any specific formal instructions, can extend composite patterns in which several mathematical functions are composed together, such as a sinusoid whose peak-to-peak amplitude increases over time. Both kindergarteners and first graders could extend such oscillating motifs correctly, not only by approximately producing the expected number of periods, but also by understanding that some functions exhibit an increasing peak-to-peak amplitude, others a decreasing one, while yet others remain constant. This finding suggests that human compositional abilities emerge much earlier than previously shown using different tasks. For example, in a task in which participants had to predict the modification of a visual item after applying two consecutive transformations on it, only 7.5 years old children (and older) succeeded (Alderete & Piantadosi, 2024). This task, however, is rather complex, since it asks participants to memorize several possible transformations on multiple visual items. Our task is more intuitive in that it asks to draw prolongations of a continuous entity (i.e., a graphical function) without requiring any training phase.

It is important to note that compositional features were less respected than other simpler characteristics such as periodicity or motif, although nearly half of the children did incorporate composition in their drawings. This reduced performance, however, does not necessarily indicate a lack of understanding of compositionality. Children may either not recognize the need to apply compositional rules or may simply favor alternative strategies that they find more appealing. Since no single correct response is expected, no approach is inherently better.

The question of when compositional abilities emerge is fundamental to theories that postulate a language-of-thought with compositional syntax in many different domains, including language, music and mathematics (Alderete & Piantadosi, 2024; Dautriche & Chemla, 2025; Dehaene et al., 2022; Frankland & Greene, 2020; Lake, Salakhutdinov, & Tenenbaum, 2015; Piantadosi et al., 2016; Pomiechowska, Bródy, Téglás, & Kovács, 2024; Sablé-Meyer et al., 2022). In this respect, in future work it would be important to test whether the emergence of compositional abilities in drawings correlates with the development of linguistic, musical and mathematical skills. According to the language of thought (LoT) hypothesis, recursively applying mental programs is an ability that lies at the heart of human singularity in all these domains. Indeed, human extrapolation of sequential spatial patterns is predicted by LoT models (Amalric et al., 2017; Mills et al., 2023) and seems to differ radically from non-human primate behavior (Dehaene, Meyniel, Wacongne, Wang, & Pallier, 2015; Mills et al., 2024; Wang, Uhrig, Jarraya, & Dehaene, 2015; Zhang et al., 2022). It seems therefore reasonable to speculate that the ability to apply mental programs to patterns and motifs should be available early on in development and lie at the origins of human geometrical and mathematical skills (Dehaene, 2026; Dehaene et al., 2022; Dehaene, Sablé-Meyer, & Ciccione, 2025). The study of children’s drawings offers an exciting new path in order to test this hypothesis.

## Supplementary Material

Supplementary data to this article can be found online at https://doi.org/10.1016/j.cognition.2025.106359.

Supplementary Material

## Data Availability

All data and scripts for data analysis are available on the OSF framework: https://osf.io/fza6u/overview.
