# Supplementary material for: Intuitions of mathematical curves in young children’s drawings
Source: Cognition. Author manuscript; Available in PMC 2025 Nov 14. (PMC7618351; doi:10.1016/j.cognition.2025.106359)
Supplement: Supplementary Material [file EMS210435-supplement-Supplementary_Material.pptx]

## Slide 1
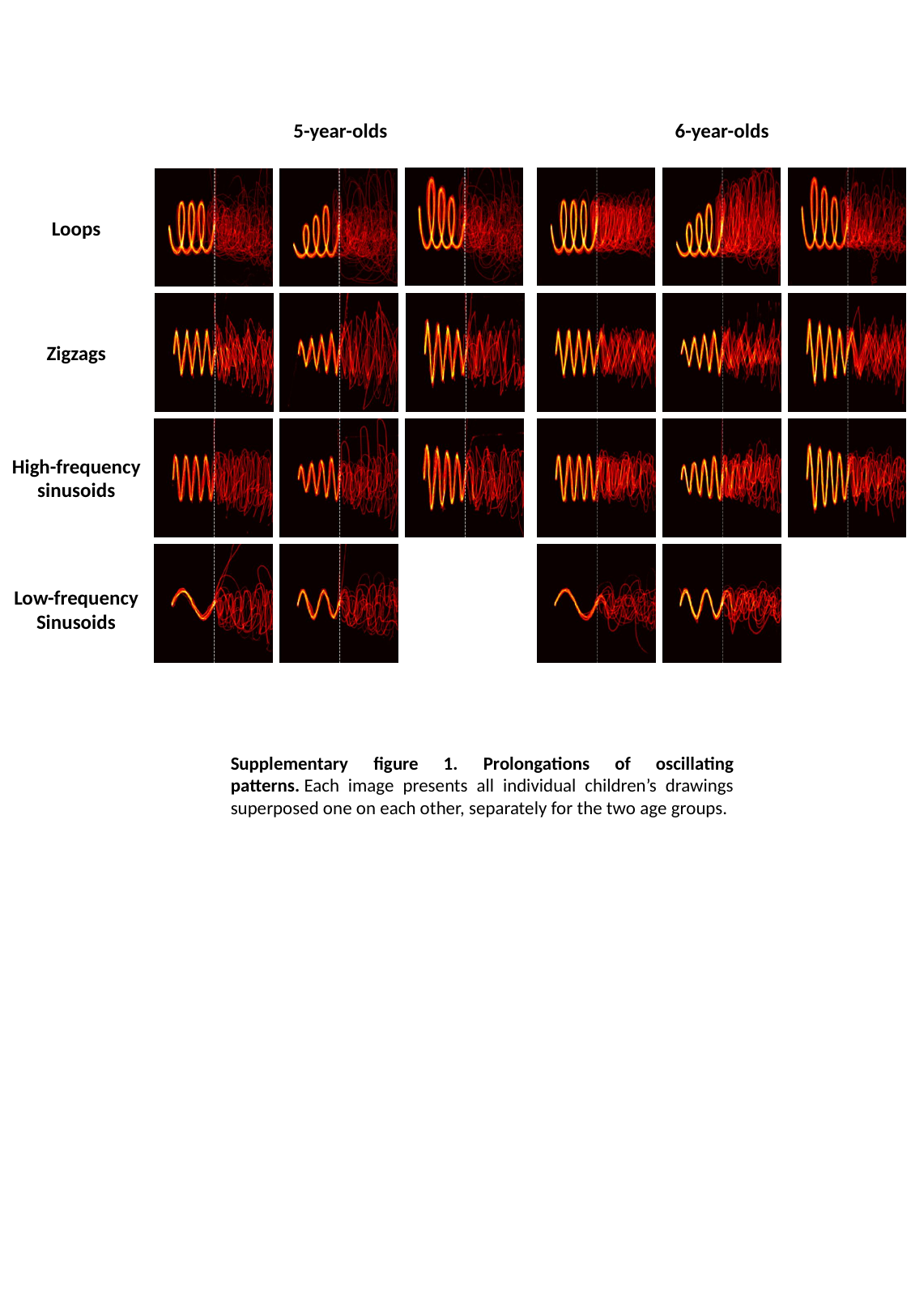

5-year-olds
6-year-olds
Loops
Zigzags
High-frequency
sinusoids
Low-frequency
Sinusoids
Supplementary figure 1. Prolongations of oscillating patterns. Each image presents all individual children’s drawings superposed one on each other, separately for the two age groups.

## Slide 2
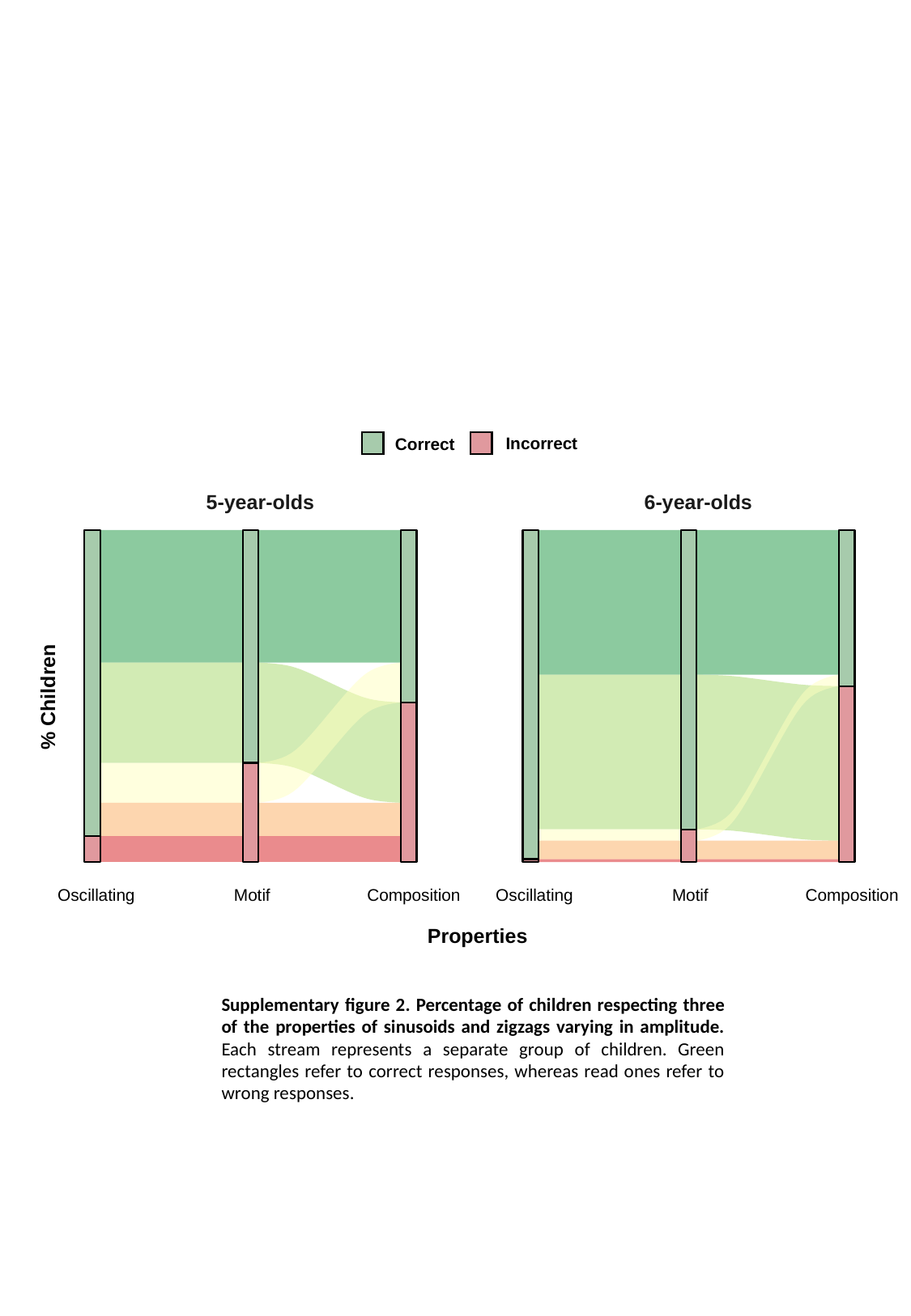

Incorrect
Correct
% Children
Oscillating
Motif
Composition
Oscillating
Motif
Composition
Properties
5-year-olds
6-year-olds
Supplementary figure 2. Percentage of children respecting three of the properties of sinusoids and zigzags varying in amplitude. Each stream represents a separate group of children. Green rectangles refer to correct responses, whereas read ones refer to wrong responses.

## Slide 3
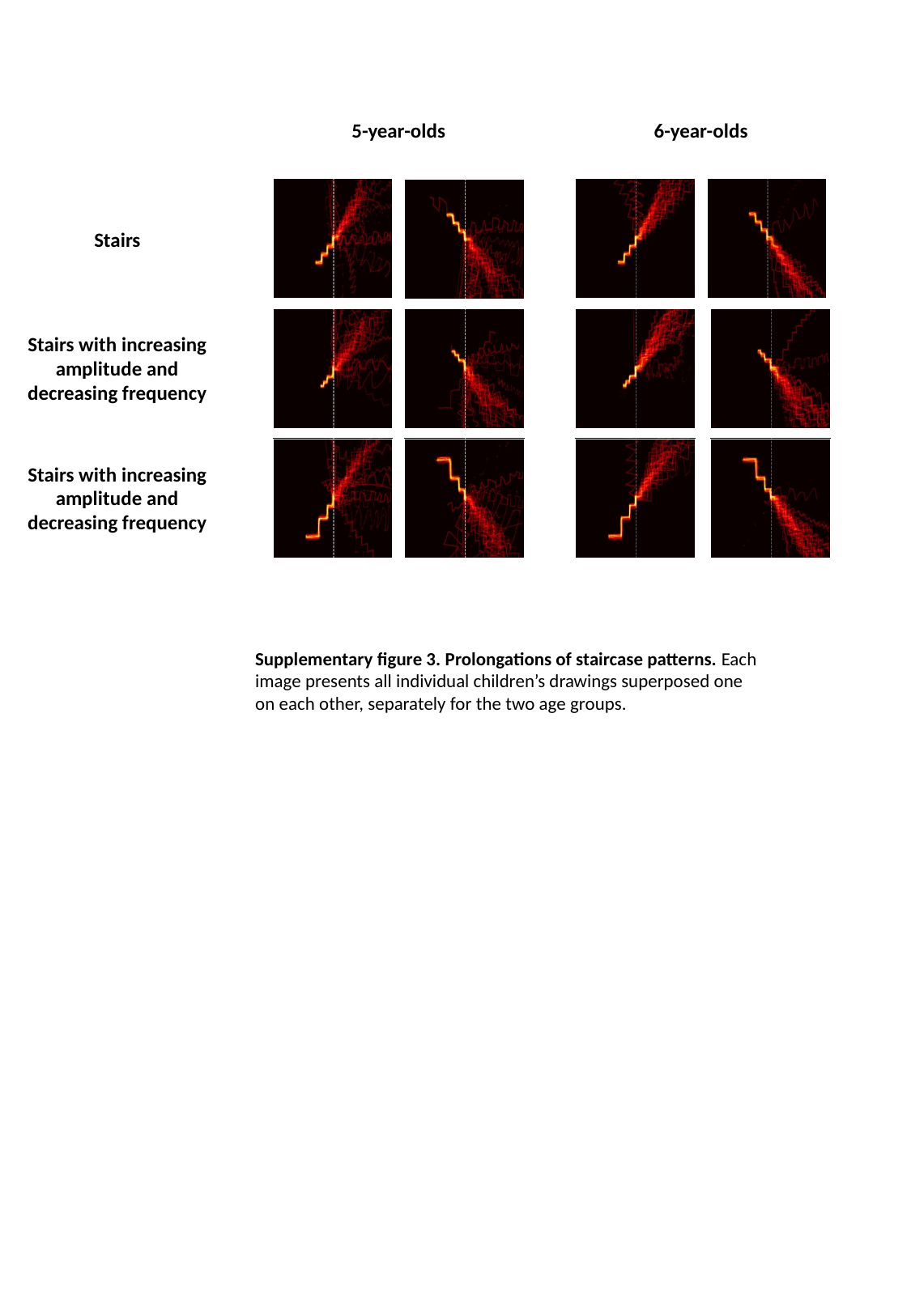

5-year-olds
6-year-olds
Stairs
Stairs with increasing amplitude and decreasing frequency
Stairs with increasing amplitude and decreasing frequency
Supplementary figure 3. Prolongations of staircase patterns. Each image presents all individual children’s drawings superposed one on each other, separately for the two age groups.

## Slide 4
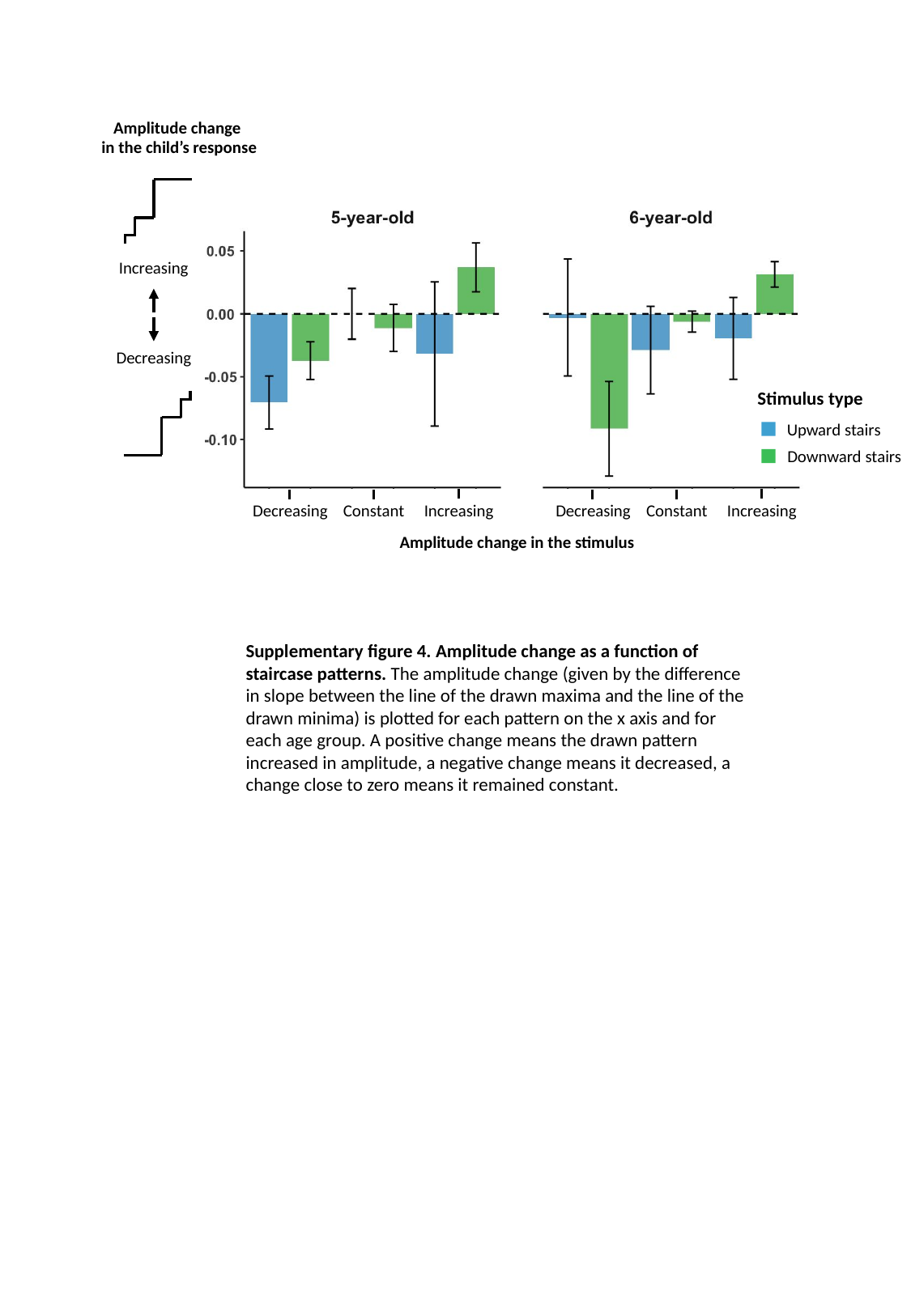

Amplitude change
in the child’s response
Increasing
Decreasing
Stimulus type
Upward stairs
Downward stairs
Decreasing
Constant
Increasing
Decreasing
Constant
Increasing
Amplitude change in the stimulus
Supplementary figure 4. Amplitude change as a function of staircase patterns. The amplitude change (given by the difference in slope between the line of the drawn maxima and the line of the drawn minima) is plotted for each pattern on the x axis and for each age group. A positive change means the drawn pattern increased in amplitude, a negative change means it decreased, a change close to zero means it remained constant.

## Slide 5
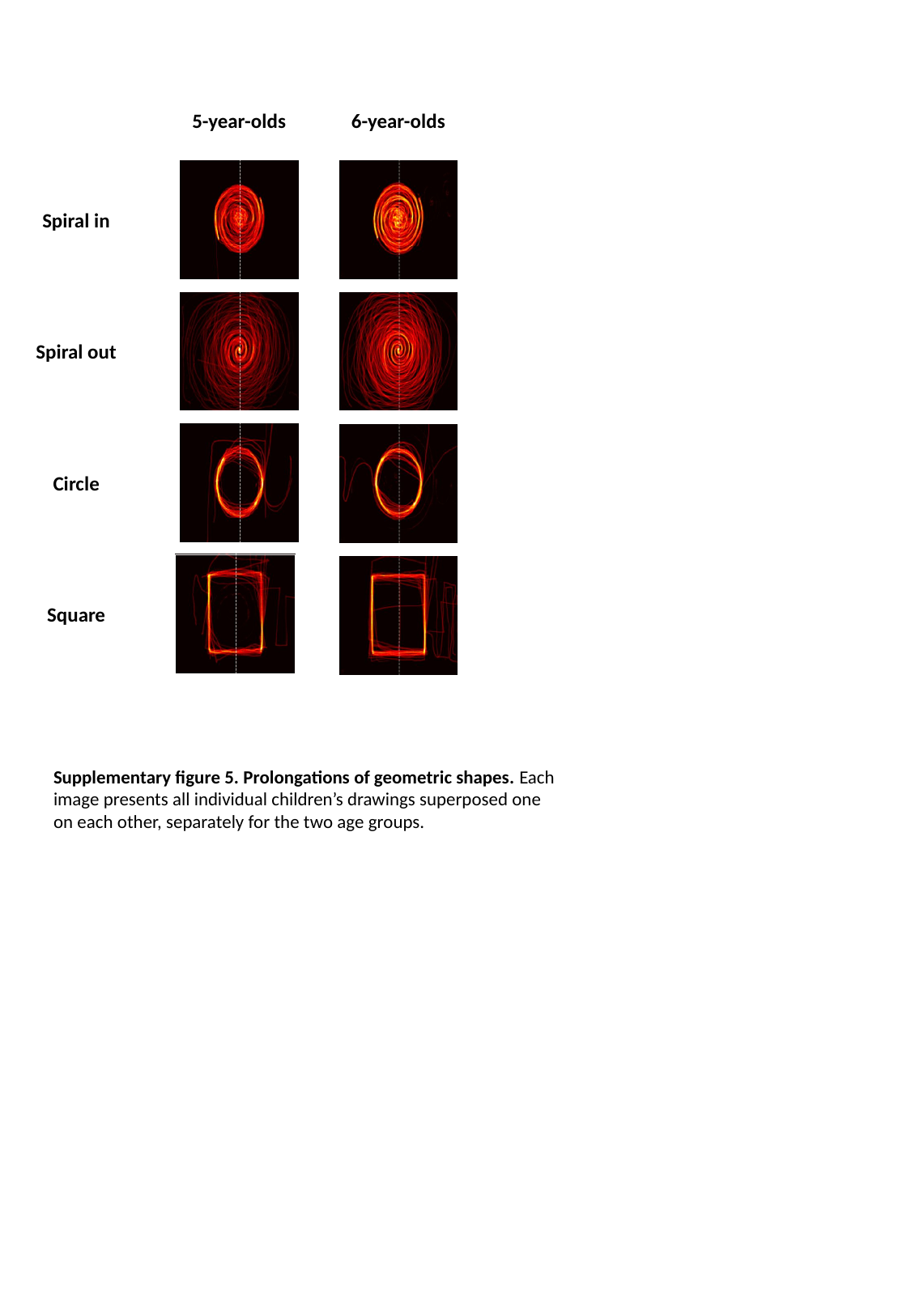

5-year-olds
6-year-olds
Spiral in
Spiral out
Circle
Square
Supplementary figure 5. Prolongations of geometric shapes. Each image presents all individual children’s drawings superposed one on each other, separately for the two age groups.

## Slide 6
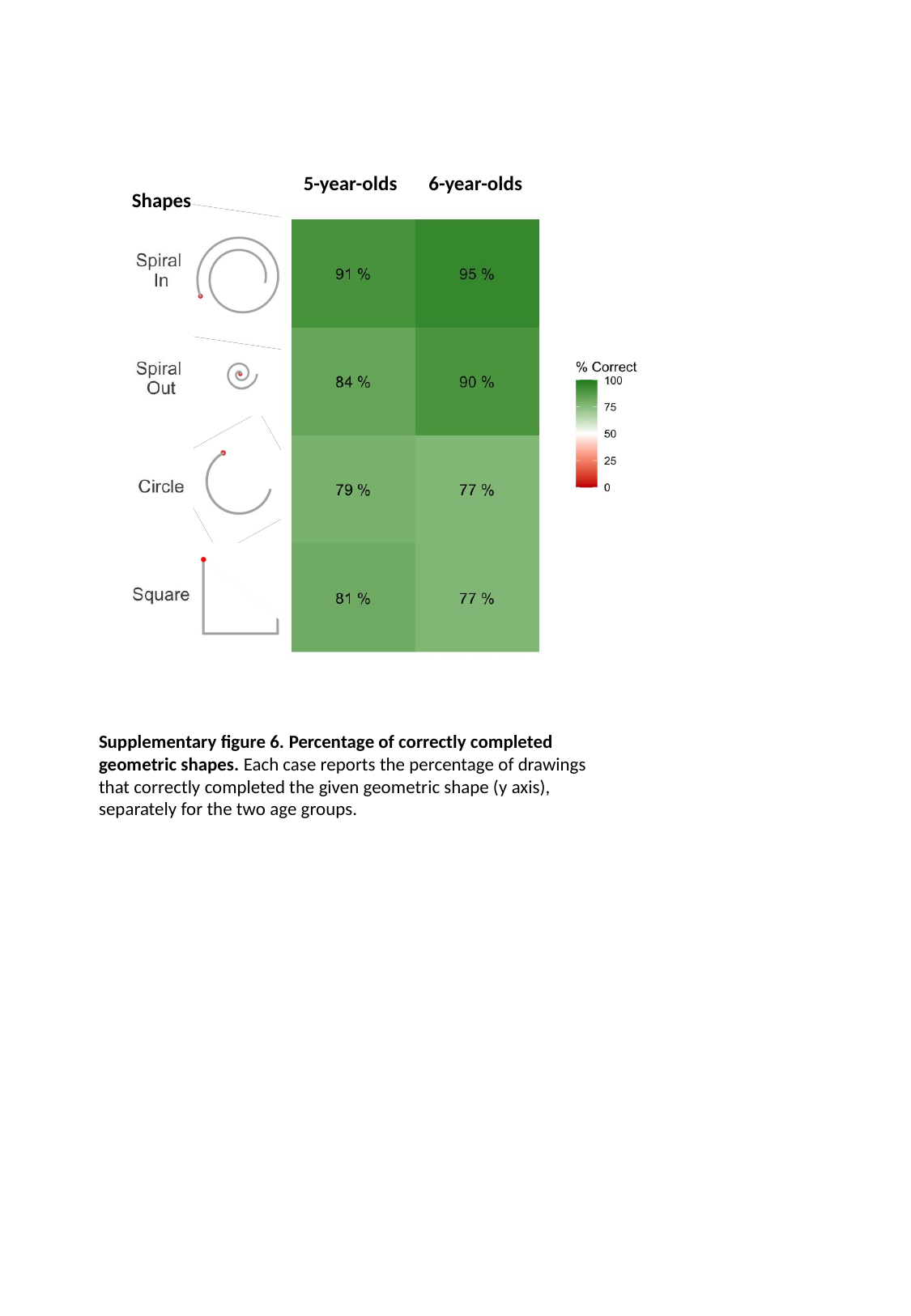

5-year-olds
6-year-olds
Shapes
Supplementary figure 6. Percentage of correctly completed geometric shapes. Each case reports the percentage of drawings that correctly completed the given geometric shape (y axis), separately for the two age groups.

## Slide 7
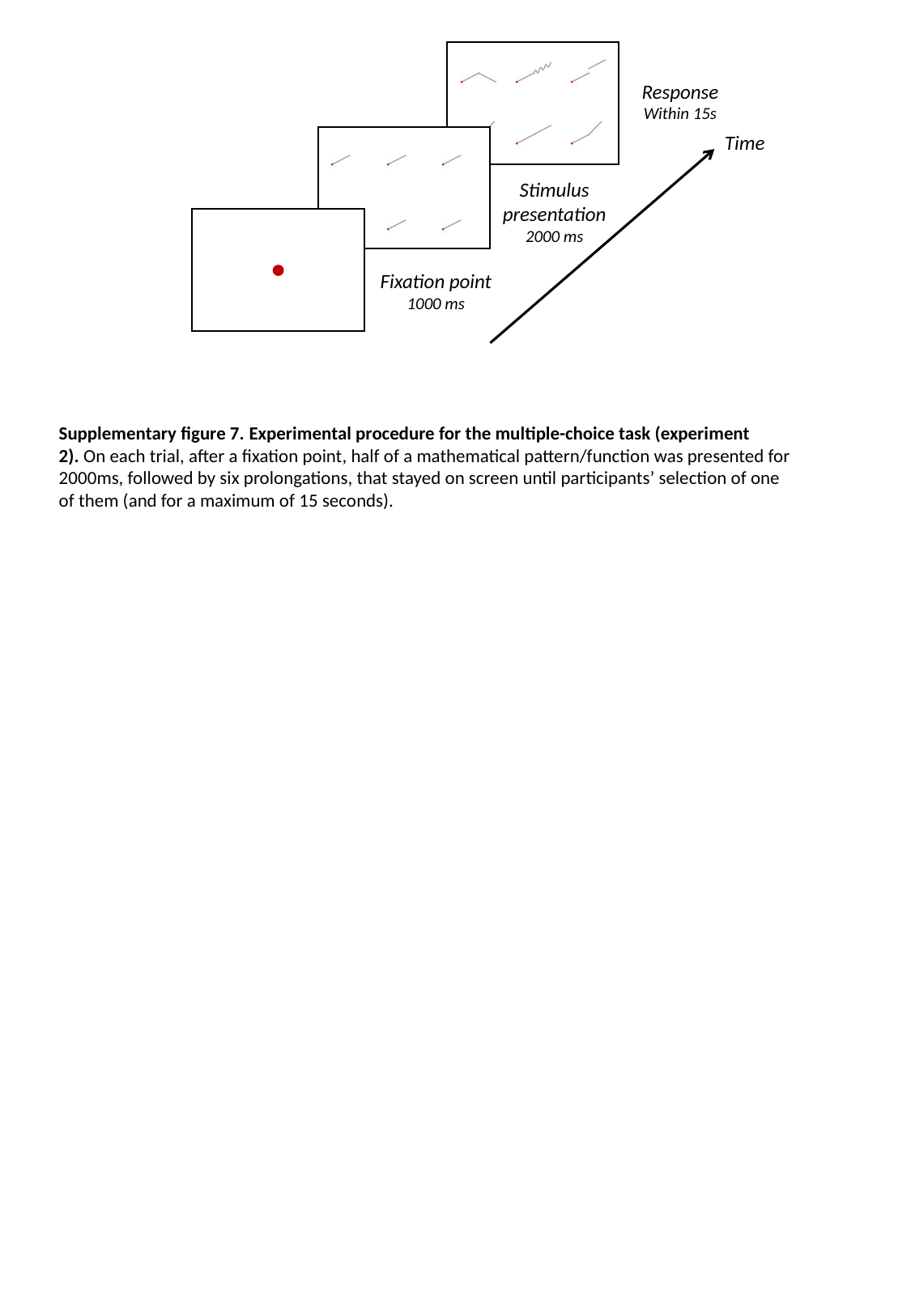

Response
Within 15s
Time
Stimulus presentation
2000 ms
Fixation point
1000 ms
Supplementary figure 7. Experimental procedure for the multiple-choice task (experiment 2). On each trial, after a fixation point, half of a mathematical pattern/function was presented for 2000ms, followed by six prolongations, that stayed on screen until participants’ selection of one of them (and for a maximum of 15 seconds).

## Slide 8
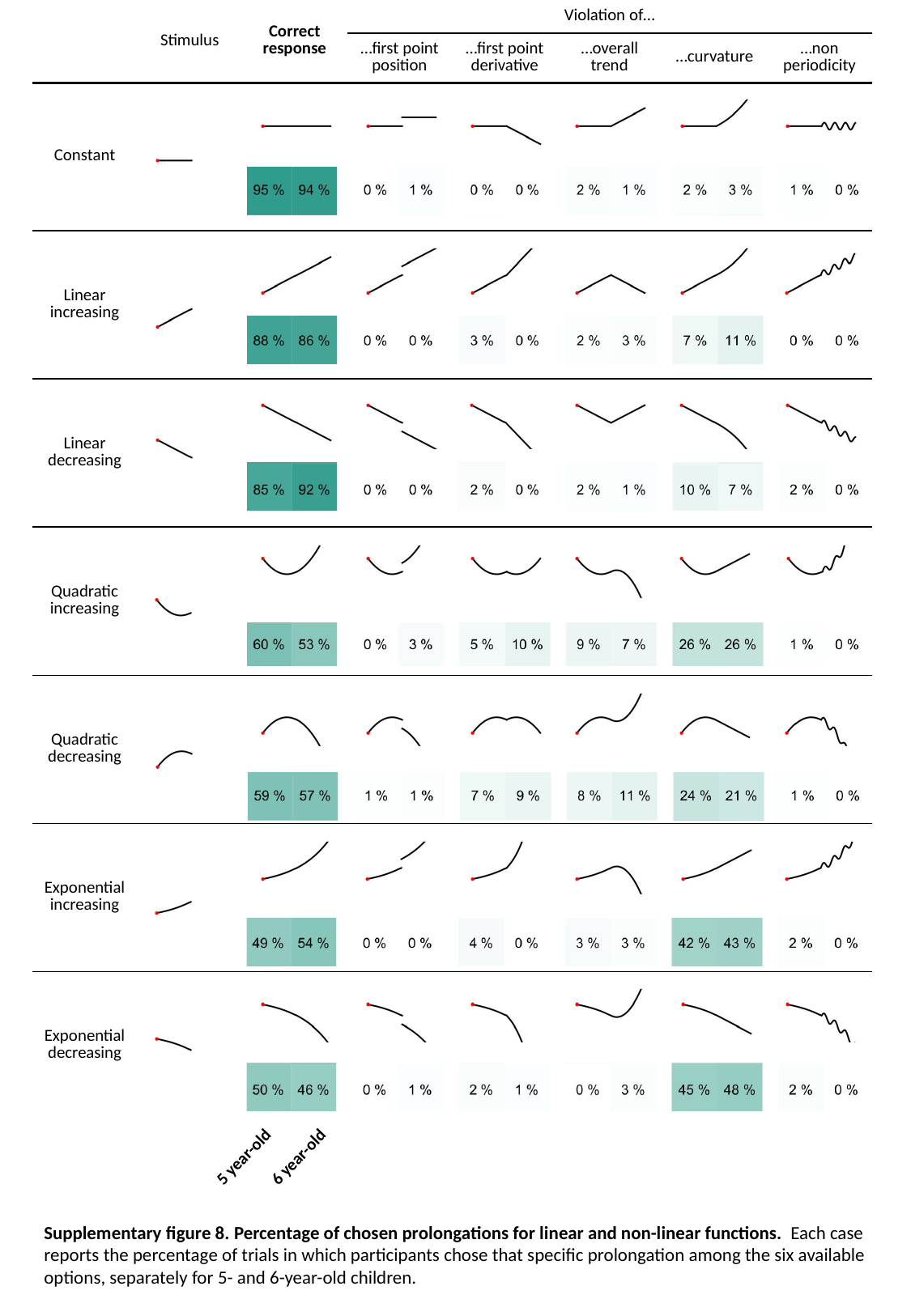

| | Stimulus | Correct response | Violation of… | | | | |
| --- | --- | --- | --- | --- | --- | --- | --- |
| | | | …first point position | …first point derivative | …overall trend | …curvature | …non periodicity |
| Constant | | | | | | | |
| Linear increasing | | | | | | | |
| Linear decreasing | | | | | | | |
| Quadratic increasing | | | | | | | |
| Quadratic decreasing | | | | | | | |
| Exponential increasing | | | | | | | |
| Exponential decreasing | | | | | | | |
5 year-old
6 year-old
Supplementary figure 8. Percentage of chosen prolongations for linear and non-linear functions.  Each case reports the percentage of trials in which participants chose that specific prolongation among the six available options, separately for 5- and 6-year-old children.

## Slide 9
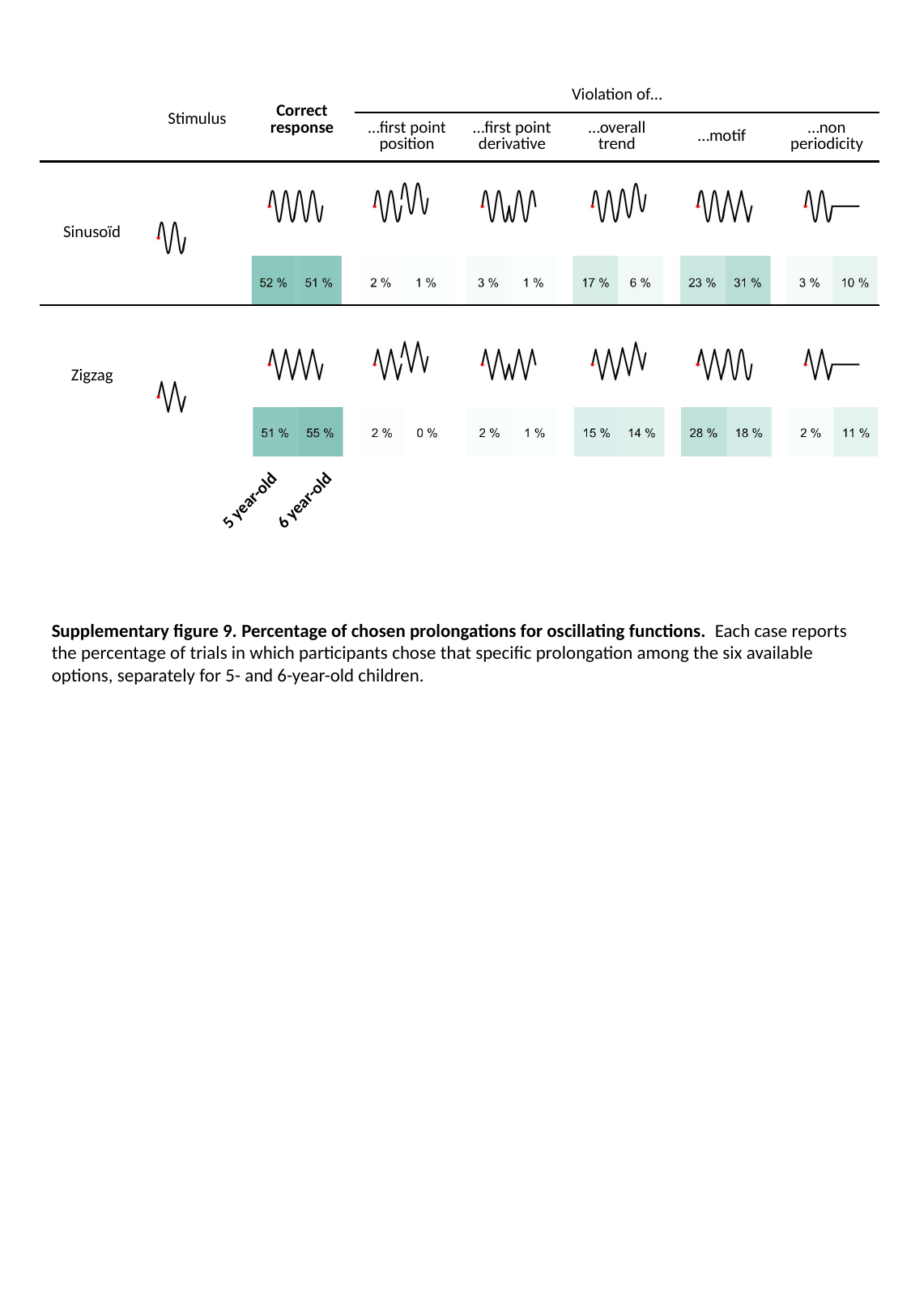

| | Stimulus | Correct response | Violation of… | | | | |
| --- | --- | --- | --- | --- | --- | --- | --- |
| | | | …first point position | …first point derivative | …overall trend | …motif | …non periodicity |
| Sinusoïd | | | | | | | |
| Zigzag | | | | | | | |
5 year-old
6 year-old
Supplementary figure 9. Percentage of chosen prolongations for oscillating functions.  Each case reports the percentage of trials in which participants chose that specific prolongation among the six available options, separately for 5- and 6-year-old children.

## Slide 10
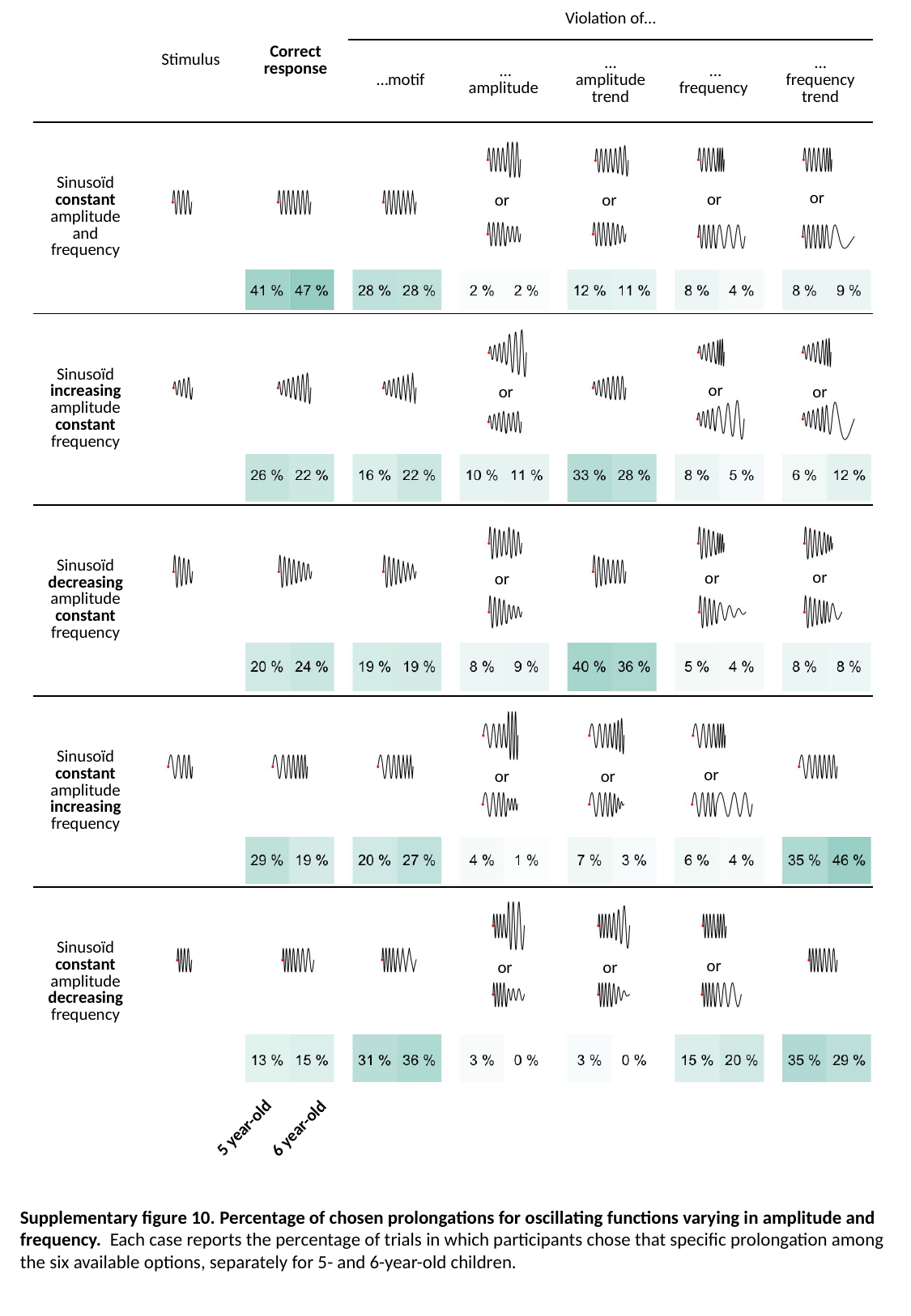

| | Stimulus | Correct response | Violation of… | | | | |
| --- | --- | --- | --- | --- | --- | --- | --- |
| | | | …motif | … amplitude | … amplitude trend | … frequency | … frequency trend |
| Sinusoïd constant amplitude and frequency | | | | | | | |
| | | | | | | | |
| Sinusoïd increasing amplitude constant frequency | | | | | | | |
| | | | | | | | |
| Sinusoïd decreasing amplitude constant frequency | | | | | | | |
| | | | | | | | |
| Sinusoïd constant amplitude increasing frequency | | | | | | | |
| | | | | | | | |
| Sinusoïd constant amplitude decreasing frequency | | | | | | | |
| | | | | | | | |
or
or
or
or
or
or
or
or
or
or
or
or
or
or
or
or
5 year-old
6 year-old
Supplementary figure 10. Percentage of chosen prolongations for oscillating functions varying in amplitude and frequency.  Each case reports the percentage of trials in which participants chose that specific prolongation among the six available options, separately for 5- and 6-year-old children.
